# The Consequences of Replicating in the Wrong Orientation: Bacterial Chromosome Duplication without an Active Replication Origin

**DOI:** 10.1128/mBio.01294-15

**Published:** 2015-11-03

**Authors:** Juachi U. Dimude, Anna Stockum, Sarah L. Midgley-Smith, Amy L. Upton, Helen A. Foster, Arshad Khan, Nigel J. Saunders, Renata Retkute, Christian J. Rudolph

**Affiliations:** a Division of Biosciences, College of Health and Life Sciences, Brunel University London, Uxbridge, United Kingdom; b Centre for Genetics and Genomics, University of Nottingham, Queen’s Medical Center, Nottingham, United Kingdom; c Department of Biochemistry, University of Oxford, Oxford, United Kingdom; d Research Institute of Health, Life and Societies, Brunel University London, Uxbridge, United Kingdom; e School of Veterinary Medicine and Science, University of Nottingham, Sutton Bonington Campus, Loughborough, United Kingdom; University of Washington; Harvard University

## Abstract

Chromosome replication is regulated in all organisms at the assembly stage of the replication machinery at specific origins. In *Escherichia coli*, the DnaA initiator protein regulates the assembly of replication forks at *oriC.* This regulation can be undermined by defects in nucleic acid metabolism. In cells lacking RNase HI, replication initiates independently of DnaA and *oriC*, presumably at persisting R-loops. A similar mechanism was assumed for origin-independent synthesis in cells lacking RecG. However, recently we suggested that this synthesis initiates at intermediates resulting from replication fork fusions. Here we present data suggesting that in cells lacking RecG or RNase HI, origin-independent synthesis arises by different mechanisms, indicative of these two proteins having different roles *in vivo*. Our data support the idea that RNase HI processes R-loops, while RecG is required to process replication fork fusion intermediates. However, regardless of how origin-independent synthesis is initiated, a fraction of forks will proceed in an orientation opposite to normal. We show that the resulting head-on encounters with transcription threaten cell viability, especially if taking place in highly transcribed areas. Thus, despite their different functions, RecG and RNase HI are both important factors for maintaining replication control and orientation. Their absence causes severe replication problems, highlighting the advantages of the normal chromosome arrangement, which exploits a single origin to control the number of forks and their orientation relative to transcription, and a defined termination area to contain fork fusions. Any changes to this arrangement endanger cell cycle control, chromosome dynamics, and, ultimately, cell viability.

**Importance** Cell division requires unwinding of millions of DNA base pairs to generate the template for RNA transcripts as well as chromosome replication. As both processes use the same template, frequent clashes are unavoidable. To minimize the impact of these clashes, transcription and replication in bacteria follow the same directionality, thereby avoiding head-on collisions. This codirectionality is maintained by a strict regulation of where replication is started. We have used *Escherichia coli* as a model to investigate cells in which the defined location of replication initiation is compromised. In cells lacking either RNase HI or RecG, replication initiates away from the defined replication origin, and we discuss the different mechanisms by which this synthesis arises. In addition, the resulting forks proceed in a direction opposite to normal, thereby inducing head-on collisions between transcription and replication, and we show that the resulting consequences are severe enough to threaten the viability of cells.

## Importance

Cell division requires unwinding of millions of DNA base pairs to generate the template for RNA transcripts as well as chromosome replication. As both processes use the same template, frequent clashes are unavoidable. To minimize the impact of these clashes, transcription and replication in bacteria follow the same directionality, thereby avoiding head-on collisions. This codirectionality is maintained by a strict regulation of where replication is started. We have used *Escherichia coli* as a model to investigate cells in which the defined location of replication initiation is compromised. In cells lacking either RNase HI or RecG, replication initiates away from the defined replication origin, and we discuss the different mechanisms by which this synthesis arises. In addition, the resulting forks proceed in a direction opposite to normal, thereby inducing head-on collisions between transcription and replication, and we show that the resulting consequences are severe enough to threaten the viability of cells.

## INTRODUCTION

**FIG 1:**
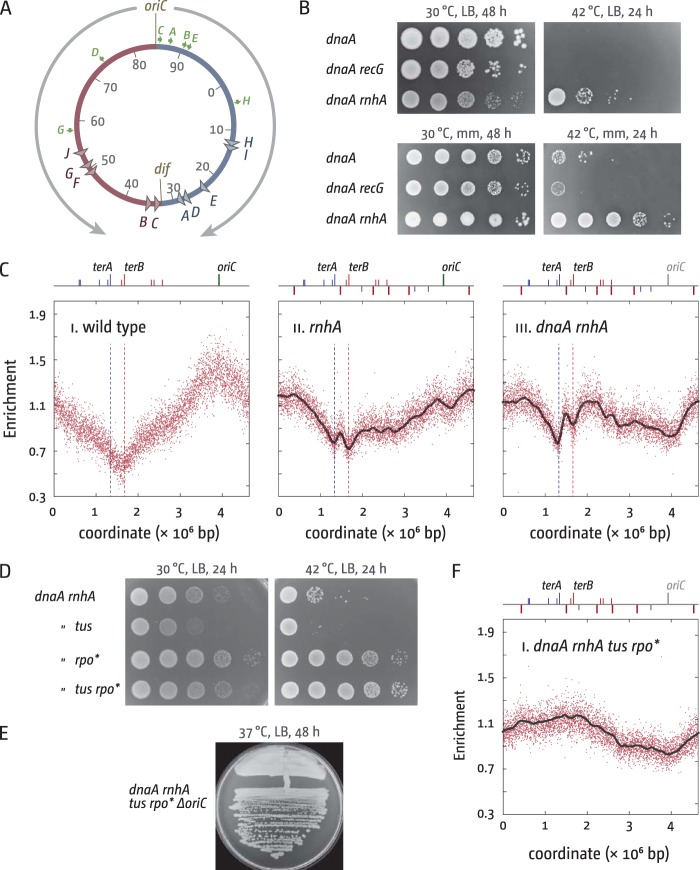
Cell growth and DNA replication in cells lacking RNase HI. (A) Schematic representation of the replichore arrangement of the *E. coli* chromosome. The normal direction of replication is indicated by gray arrows. *ter* sites (triangles) are identified by their corresponding letter (e.g., “A” indicates *terA*). The numbers represent the minutes of the standard genetic map (0 to 100 min). The origin of replication (*oriC*) and the chromosomal dimer resolution site (*dif*) are marked. The green arrows represent the location and direction of transcription of the 7 *rrn* operons *rrnA* to *E*, *rrnG*, and *rrnH*. (B) Spot dilution assay to evaluate origin-independent growth in *dnaA*, *dnaA recG*, and *dnaA rnhA* cells on LB agar and minimal medium. The strains used were AU1054 (*dnaA46*), AU1091 (*dnaA recG*), and AU1066 (*dnaA rnhA*). (C) Marker frequency analysis of *E. coli* cells in the exponential phase. The number of reads (normalized against a stationary-phase wild-type control) is plotted against the chromosomal location. The 5 main (red) and 3 minor (purple) origin-independent initiation sites, as well as the positions of *oriC* (green line) and all *ter* sites, are shown above the plotted data, with red and blue lines representing the left and right replichores, respectively. A gray loess regression curve (see Text S1 in the supplemental material) is shown for the *rnhA* (ii) and *dnaA rnhA* (iii) cells. Sequencing templates were isolated from MG1655 (wild type), N4704 (*rnhA*), and AU1066 (*dnaA rnhA*). All cultures were grown at 42°C. (D) Spot dilution assay showing the effect of *tus* and rpo* mutations on growth of *dnaA rnhA* cells. The strains used were AU1066 (*dnaA46 rnhA*), RCe218 (*dnaA46 rnhA tus*), RCe303 (dnaA46 rnhA rpo*), and RCe309 (dnaA46 rnhA tus rpo*). (E) rnhA tus rpo* cells can tolerate deletion of the entire *oriC* region (RCe395). (F) Marker frequency analysis of dnaA rnhA tus rpo* cells grown at 42°C. Sequencing templates were isolated from RCe309 (dnaA rnhA tus rpo*).

Chromosome duplication is regulated by the recruitment of the replication machinery to specific initiation sites (origins) where two forks are established and move in opposite directions until they meet either an opposing fork or the end of a chromosome. In *Escherichia coli*, DnaA protein controls replication initiation of the circular chromosome at *oriC* (1). Two replisomes are recruited and proceed in opposite directions until they meet within a specialized termination zone opposite *oriC*. This zone is flanked by *ter* sequences (*terA* to *J*), which, if bound by Tus protein, form polar traps that restrict fork movement (2). This divides the chromosome into two replichores—one replicated by the fork moving clockwise, the other by the fork moving counterclockwise (3). The majority of highly transcribed genes within each replichore are oriented in the same direction as replication, thereby minimizing head-on collisions between the transcription and replication machineries, which were suggested to be particularly problematic (4–7) (Fig. 1A).

Replication can initiate independently of DnaA and *oriC*. This origin-independent or stable DNA replication (SDR) is observed in cells with defects in nucleic acid metabolism. In cells lacking RNase HI (encoded by the *rnhA* gene), SDR is sufficiently robust to sustain growth independently of *oriC* firing, especially on minimal agar (8–10) (Fig. 1B). Thus, *rnhA* cells carrying a temperature-sensitive *dnaA46* allele (referred to as *dnaA* in the rest of the text) grow at a restrictive temperature of 42°C (Fig. 1B). Because RNase HI removes RNA from DNA:RNA hybrid duplexes (11), Kogoma and coworkers suggested that SDR initiates at R-loops (8). Similarly, RecG protein can unwind the RNA from R-loops (12, 13), and *recG* cells also show increased levels of SDR (14, 15), suggesting that SDR in both backgrounds might have a common underlying mechanism (8, 16).

Recently we published results indicating that RecG helicase is a key player for processing intermediates arising from the fusion of two replication forks (10, 15, 17, 18). Replication profiles determined by high-resolution marker frequency analysis (19, 20) revealed that the vast majority of SDR in exponentially growing *recG* single mutants occurs in the termination area (10). Our genetic and cell biology data are consistent with the idea that fusion of two approaching replisomes can result in the formation of a 3′ flap. 3′ flaps are normally processed by RecG or 3′ exonucleases. However, in the absence of RecG, a fraction of these 3′ flaps persist and are processed instead by PriA, which triggers the formation of additional replication forks, which then start to rereplicate the already replicated DNA (10). Normally, this overreplication is contained by the *ter*/Tus replication fork trap, explaining why temperature-sensitive *dnaA* [*dnaA*(Ts)] *recG* cells are unable to grow at restrictive temperature (Fig. 1B). Upon inactivation of the replication fork trap by deletion of *tus*, synthesis can proceed, but it does so against the normal orientation of replication, resulting in conflicts with ongoing transcription (10). These conflicts can be alleviated by an rpoB*35 point mutation, which reduces the ability of transcribing RNA polymerase complexes to pause and backtrack (21), thereby reducing the conflicts between replication and transcription (21, 22). Indeed, recG tus rpoB*35 cells show robust growth in the absence of origin firing and can tolerate deletion of the entire *oriC* region (10).

In this study, we present evidence demonstrating that the mechanism for origin-independent synthesis in cells lacking RNase HI differs from that operating in cells lacking RecG. Our data support the suggestion that SDR in cells lacking RNase HI is mostly initiated at R-loops located at various sites along the chromosome. In contrast, R-loops do not appear to play a major role in the origin-independent synthesis in *recG* cells, where the majority of synthesis appears to be triggered by the formation of 3′ flaps at replication fork fusion sites. However, regardless of how origin-independent synthesis is initiated in both backgrounds, a significant number of the resulting forks proceed in the opposite direction from that normally dictated by the replichore arrangement, leading to more frequent head-on encounters with ongoing transcription complexes. These replication-transcription encounters threaten cell viability. Thus, both RecG and RNase HI are important factors for maintaining control of replication initiation, replication orientation, and limitation of fork collision events, and the absence of either causes serious problems in cell cycle control, chromosome dynamics, and genomic stability.

## RESULTS

Initiation of chromosome replication is normally precisely regulated by the DnaA initiator protein and restricted to the *oriC* area (23). However, in the absence of RNase HI, a number of additional initiation sites away from *oriC* were shown by Kogoma’s laboratory, including a cluster in the termination area (8, 24). Even though termed *oriK*s, these areas are not origins in the conventional sense, as they are not able to maintain plasmid replication if cloned into a plasmid without a functional origin in an *rnhA* background (25). Recently Maduike et al. published high-resolution marker frequency analyses by deep sequencing in cells lacking RNase HI (9). We have conducted similar experiments (Fig. 1C). For each strain examined, we established a replication profile based on the ratio of uniquely mapped sequence reads in a replicating sample to a nonreplicating control sample (stationary-phase wild-type cells) sequenced in parallel. The peaks observed in our profiles are more pronounced but otherwise in good agreement with those from previous studies (9, 24), suggesting that initiation of synthesis takes place in at least five chromosomal locations, as suggested in reference 9. A loess regression curve (see Text S1 in the supplemental material) suggests 5 main initiation sites at 0.4, 1.47, 2.24, 2.60, and 4.54 Mbp besides *oriC*, with potentially minor additional initiation sites at 1.98, 3.2, and 3.5 Mbp (Fig. 1C). This increased number of initiation sites is likely to be responsible for the replication profile of *rnhA* cells being flatter than the profile of wild-type cells. Indeed, it was shown before that recombination-dependent replication initiation in multiple locations in *Haloferax volcanii* cells lacking all replication origins led to a completely flat replication profile (26).

In contrast, we demonstrated recently that in cells lacking RecG, the majority of origin-independent synthesis is initiated in the termination area (10, 17), and as described in detail in the introduction, we suggested that this overreplication is the result of intermediates accumulating when replication forks fuse (10). This overreplication is efficiently blocked by the *ter*/Tus replication fork trap but can proceed into the replichores upon deletion of *tus*. However, it does so against the normal orientation of replication, which results in conflicts with ongoing transcription (10). These conflicts can be alleviated by an rpoB*35 point mutation (10), which reduces the stability of transcribing RNA polymerase complexes (6, 21).

As synthesis is also initiated within the termination area of *rnhA* cells, we investigated the effect of a *tus* deletion. The absence of Tus in *dnaA rnhA* cells caused a mild reduction of growth at both 30°C and 42°C (Fig. 1D), suggesting perhaps that the release of synthesis from the termination area causes more harm than good. In contrast, introduction of an rpo* point mutation improved growth quite significantly in both *dnaA rnhA* and *dnaA rnhA tus* cells (Fig. 1D) and allowed the deletion of the entire *oriC* region (Fig. 1E). Marker frequency analysis of dnaA rnhA tus rpo* cells grown at the restrictive temperature revealed a relatively flat but effectively inverted replication profile (Fig. 1F), in which the major peaks observed in *dnaA rnhA* cells (Fig. 1C) are fused to a broadly elevated region roughly between 0.5 and 2.6 Mbp, while the *oriC* region shows a distinctly low marker frequency level (described below). These results are in line with the idea that a proportion of replication forks in *dnaA rnhA* cells initiated away from *oriC* will proceed in an orientation opposite to normal, thereby inducing conflicts with transcription. From these results, we conclude that the reported broth sensitivity of *dnaA rnhA* cells (Fig. 1B and D) (8) is, at least in part, caused by replication-transcription conflicts.

### The genetic requirements for origin-independent synthesis in the termination area differ in cells lacking RecG and RNase HI.

Both cells lacking RecG and those lacking RNase HI show a peak of synthesis in the termination area (Fig. 1C) (10), opening the question whether this synthesis arises by a similar mechanism (16). We showed before that initiation of origin-independent synthesis in *recG* cells has distinct genetic requirements, as it was dependent on PriA helicase activity (10). Even more specifically, it was entirely dependent on the specific ability of PriA helicase to process 3′ flap structures (10). The *srgA1* allele of *priA* encodes a mutant protein (PriA L557P) with a very specific alteration in its biochemical substrate specificity. It unwinds a replication fork with both a leading strand and a lagging strand at the branch point as efficiently as wild-type PriA, but it has lost the ability to unwind a fork in which the leading strand is missing (27), the equivalent of a 3′ flap. This led us to suggest that a 3′ flap structure persists in the absence of RecG, a result supported by the finding that the lack of 3′ exonucleases also results in overreplication in the termination area (10, 28).

**FIG 2:**
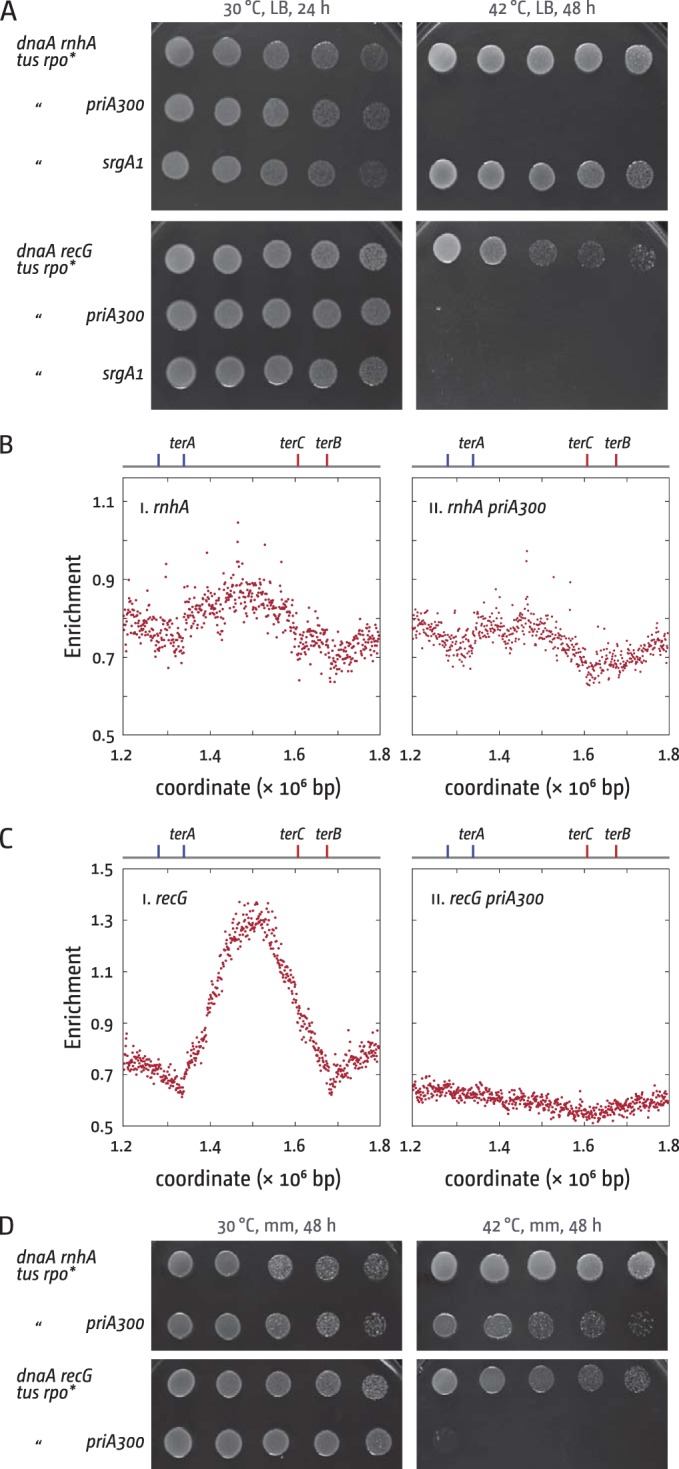
Effect of PriA helicase mutations on origin-independent synthesis in *rnhA* and *recG* cells. (A) Spot dilution assays to evaluate origin-independent growth in dnaA rnhA tus rpo* and dnaA recG tus rpo* cells in the absence of PriA helicase activity on LB agar. The strains used were RCe309 (dnaA rnhA tus rpo*), JD1162 (dnaA rnhA tus rpo* priA300), JD1163 (dnaA rnhA tus rpo* srgA1), RCe268 (dnaA recG tus rpo*), RCe313 (dnaA recG tus rpo* priA300), and JD1167 (dnaA recG tus rpo* srgA1). (B and C) Marker frequency analysis of the termination area of *E. coli* cells in the exponential phase. The number of reads (normalized against the reads for a stationary-phase wild-type control) is plotted against the chromosomal location. Positions of *ter* sites in the area are highlighted. Sequencing templates were isolated from AM1975 (*rnhA*) and JD1085 (*rnhA priA300*). Data for *recG* (N6576) and *recG priA300* (N6859) cells were replotted from reference 10. (D) Spot dilution assays to evaluate origin-independent growth in dnaA rnhA tus rpo* and dnaA recG tus rpo* cells in the absence of PriA helicase activity on minimal agar. The strains used were RCe309 (dnaA rnhA tus rpo*), JD1162 (dnaA rnhA tus rpo* priA300), RCe268 (dnaA recG tus rpo*), and RCe313 (dnaA recG tus rpo* *priA300*).

We introduced the *srgA1* allele into a dnaA rnhA tus rpo* background and found that the cells grow robustly at 42°C, much in contrast to dnaA recG tus rpo* srgA1 cells (Fig. 2A). Thus, the specific substrate activity of PriA helicase is essential for the initiation of synthesis in cells lacking RecG but not in cells lacking RNase HI, suggesting that different structures accumulate in the absence of each of these proteins. Introduction of a *priA300* allele, which encodes the helicase-deficient PriA K230R (29), into a dnaA rnhA tus rpo* background reduced growth at 42°C on LB agar substantially, as observed previously (8, 30) and similar to the reduced growth seen in dnaA recG tus rpo* priA300 cells (Fig. 2A) (10). However, the replication profile of *rnhA priA300* cells grown in LB broth revealed that the overreplication in the termination area was not totally abolished but only mildly reduced (Fig. 2B), much in contrast to *recG priA300* cells where no trace of overreplication can be found (Fig. 2C) (10). In line with this result, we were able to demonstrate growth of dnaA rnhA tus rpo* priA300 cells on minimal medium, in contrast to dnaA recG tus rpo* priA300 cells (Fig. 2D). Thus, it appears that the helicase activity of PriA is not absolutely essential for origin-independent synthesis in cells lacking RNase HI, in line with the idea that the DNA intermediates accumulating in cells lacking RecG and RNase HI are different.

**FIG 3:**
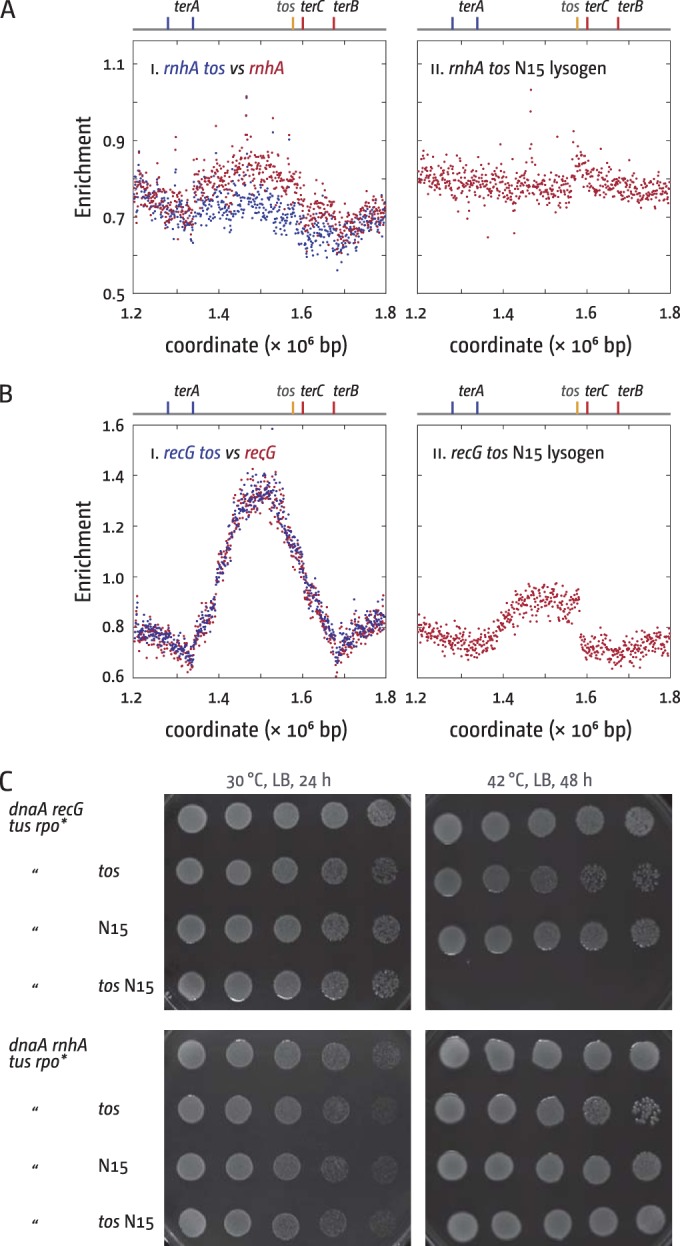
Effect of chromosome linearization on origin-independent synthesis in *recG* and *rnhA* cells. (A and B) Marker frequency analysis of the termination area of *E. coli* cells in the exponential phase. The number of reads (normalized against the reads for a stationary-phase wild-type control) is plotted against the chromosomal location. Positions of *ter* sites in the area as well as the integration site of the *tos* linearization sequence are highlighted. In panels Bi and Ci, data sets for *recG* and *recG tos* as well as *rnhA* and *rnhA tos* are plotted together for direct comparison. Sequencing templates were isolated from AM1975 (*rnhA*), RCe605 (*rnhA tos*), and RCe608 (*rnhA tos* N15 lysogen). Data for *recG* (N8226), *recG tos* (RCe391), and *recG tos* N15 lysogen (RCe399) were replotted from reference 10. (C) Spot dilution assays to evaluate DnaA-independent growth in *dnaA rnhA* cells with a linearized chromosome. The strains used were RCe309 (dnaA46 rnhA tus rpo*), JD1160 (dnaA46 rnhA tus rpo* tos), JD1168 (dnaA46 rnhA tus rpo* N15 lysogen), JD1169 (dnaA46 rnhA tus rpo* tos N15 lysogen), RCe268 (dnaA46 recG tus rpo*), RCe385 (dnaA46 recG tus rpo* tos), RCe384 (dnaA46 recG tus rpo* N15 lysogen), and RCe387 (dnaA46 recG tus rpo* tos N15 lysogen).

In addition, we found that chromosome linearization, which prevents the collision of replisomes, has different effects in cells lacking RecG or RNase HI. Linearization is achieved by introducing the *tos* linearization sequence from bacteriophage N15 near the chromosome dimer resolution site *dif* into the chromosome. Subsequent lysogenic infection with N15 results in the expression of the telomerase TelN, which will process the *tos* linearization sequence (31). We found that insertion of the *tos* site in *rnhA* cells resulted in a mild reduction of the overreplication in the termination area, a result not observed in *recG* cells (cf. Fig. 3Ai and 3Bi). Thus, it appears that integration of the *tos* linearization cassette interferes with initiation of overreplication in the termination area in *rnhA* but not *recG* cells. Linearization of the chromosome, which is clearly visible as a discontinuity in the replication profile (Fig. 3A and B; see Fig. S1 in the supplemental material) (19), caused a reduction of overreplication, both in cells lacking RecG and those lacking RNase HI. However, the resulting profiles look different. Upon linearization, the profile of *rnhA* cells becomes asymmetric, with no overreplication observed between *tos* and *terA*, while some overreplication is observed between *terC*/*B* and *tos*, which appears abruptly cut off at the linearization site (Fig. 3Aii). In contrast, *recG* cells with a linearized chromosome show elevated marker frequency levels on both sides of the linearization site (Fig. 3Bii).

To investigate the effect of chromosome linearization on the ability of *rnhA* and *recG* cells to grow in the absence of origin firing, we linearized the chromosome in dnaA recG tus rpo* and dnaA rnhA tus rpo* cells. As shown in Fig. 3C, chromosome linearization showed no detectable effect on the growth of dnaA rnhA tus rpo* cells, much in contrast to dnaA recG tus rpo* cells, as reported previously (10). This suggests that the overreplication in the termination area in *recG* cells is responsible for the ability of dnaA recG tus rpo* cells to grow at 42°C. In contrast, for growth of dnaA rnhA tus rpo* cells, synthesis in the termination area is completely dispensable.

### Origin-independent synthesis outside the termination area.

Origin-independent synthesis in *dnaA recG* cells, while prevalent in the termination area, is observed in all chromosomal areas at a low level (17), and while synthesis at the terminus is reduced upon chromosome linearization, synthesis elsewhere is not (10). Thus, origin-independent synthesis in *recG* cells might arise by two independent mechanisms. The absence of growth of dnaA recG tus rpo* cells with a linearized chromosome already indicates that the synthesis observed outside the termination area is not strong enough to allow the formation of visible colonies. However, it might allow abortive growth.

**FIG 4:**
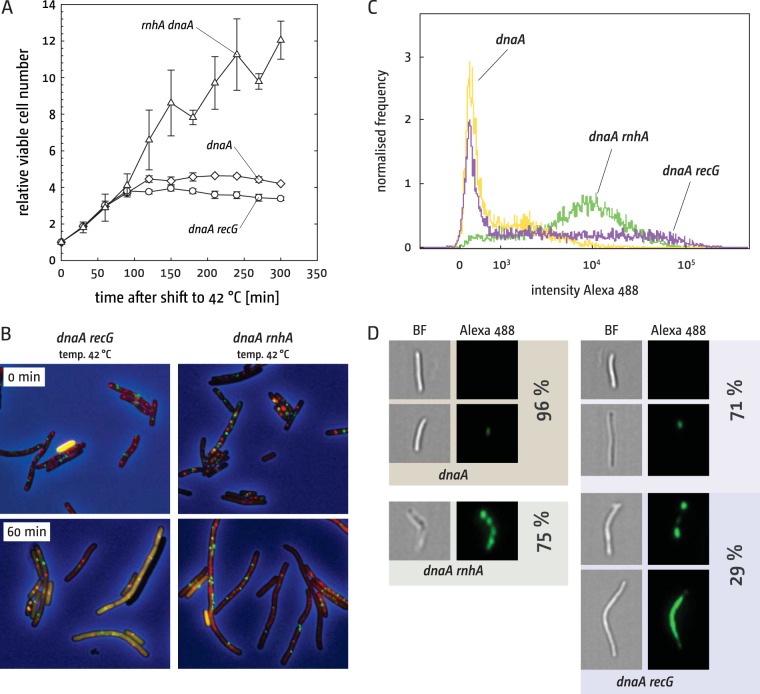
Cell replication and chromosome dynamics in cells lacking RNase HI. (A) Viable cell counts of *dnaA* derivatives following shift to restrictive temperature. Cells were grown at the permissive temperature to early exponential phase and shifted to 42°C, and samples were taken at the times indicated. Samples were diluted, plated, and incubated at the permissive temperature. The strains used were AU1054 (*dnaA46*), AU1091 (*dnaA recG*), and AU1066 (*dnaA rnhA*). (B) Fluorescence microscopy shows replication of origin (red foci) and terminus (green foci) areas of the chromosome. (Combined phase-contrast and fluorescence images are shown.) The strains used were RCe198 (*dnaA recG*) and RCe202 (*dnaA rnhA*). Incubation times following the shift to 42°C are indicated. (C) Imaging flow cytometry analysis of EdU incorporation into newly synthesized DNA, followed by Alexa Fluor 488 click labeling. The strains used were AU1054 (*dnaA46*), AU1091 (*dnaA recG*), and AU1066 (*dnaA rnhA*). The strains were shifted to 42°C for 90 min to inhibit *oriC* firing without affecting ongoing synthesis. All strains were labeled with EdU for 15 min before fixing and click labeling. The histogram shows the fraction of cells against fluorescence intensity. A minimum of 5,000 in-focus cells were analyzed for each strain. The experiment from which the data are plotted was repeated with a similar number of cells and was reproducible. (D) Images of cells are representative for fluorescence levels, and the distribution in the genetic backgrounds indicated was derived from the imaging flow cytometry data collection. Images of bright-field and Alexa Fluor 488 fluorescence are shown at 60× magnification. The percentages of cells with the shown fluorescence levels are indicated.

To investigate whether synthesis outside the termination area can contribute to cell duplication, we directly followed the viable titer of *dnaA*, *dnaA recG*, and *dnaA rnhA* cells following shift to 42°C. Both *dnaA* and *dnaA recG* cells showed approximately 2 cell division events in LB broth before growth arrest (Fig. 4A), with no hint of further divisions. In contrast, *dnaA rnhA* cells showed continuous growth over several hours (Fig. 4A). Growth is linear rather than exponential, which is likely to be caused by the broth sensitivity of *dnaA rnhA* cells (Fig. 1B) (8), and *dnaA rnhA* cells showed robust levels of bromodeoxyuridine (BrdU) incorporation in all chromosomal areas (see Fig. S2A in the supplemental material). Thus, synthesis in *dnaA rnhA* cells can contribute toward successful cell duplication for significant periods of time, whereas synthesis outside the termination area in *recG* cells cannot.

We used fluorescent repressor-operator arrays in the origin and terminus area of the chromosome to follow chromosome dynamics in *dnaA* cells lacking either RecG or RNase HI (Fig. 4B). We previously reported that a *dnaA* background forms cells with single origin and terminus foci 120 min after shift to restrictive temperature (15). We analyzed focus counts in the previously obtained images (15) and found that 60 min following the shift to 42°C, the majority of cells showed a single origin and terminus focus (65%), indicative of a single copy of a fully replicated chromosome. An additional 18% showed 2 origin and 2 terminus foci, suggesting that the presence of two fully replicated chromosomes without septation having taken place yet.

In line with the observed arrest in cell growth (Fig. 4A), 75% of *dnaA recG* cells showed either a single origin and terminus focus or 2 separated chromosomes 60 min following a shift to 42°C (Fig. 4B). However, we observed an increased number of cells with aberrant focus counts. Twenty percent of *dnaA recG* cells showed 3 or more terminus foci (6% in *dnaA* single mutants), and 7% showed 8 or more origin foci (2.5% in *dnaA* single mutants). This uncontrolled amplification of chromosomal areas is typical for *recG* cells suffering from genotoxic damage (15).

The situation proved different in cells lacking RNase HI (Fig. 4B). While the focus distribution in *dnaA rnhA* cells growing at 30°C was overall similar to that in *dnaA* cells, we observed an increased number of *dnaA rnhA* cells in which the number of terminus foci exceeded the number of origin foci (15%), in line with synthesis initiating at multiple sites in and near the terminus region (Fig. 1C) (9, 24). Sixty minutes after the shift to 42°C, only a small fraction of cells showed a single origin and terminus focus (6%). Instead, the number of cells in which the number of terminus foci exceeded the number of origin foci was increased to 37%, with 34% showing more than 3 terminus foci per cell (Fig. 4B).

Thus, the majority of *dnaA* and *dnaA recG* cells arrest growth upon the shift to 42°C, with a fraction of cells showing a significant amplification of limited chromosomal areas, presumably because they suffer from some form of spontaneous DNA damage (17). To establish how much this fraction of cells contributes to the overall synthesis visualized by BrdU incorporation, we visualized newly replicated DNA via pulse-labeling with 5-ethynyl-2′-deoxyuridine (EdU) (32). *dnaA*, *dnaA recG*, and *dnaA rnhA* cells were shifted to 42°C for 90 min to stop *oriC* firing while allowing ongoing forks to complete synthesis. Cells were then pulse-labeled with EdU for 15 min and visualized via high-resolution microscopy in flow with an Amnis ImageStream^x^ Mark II. As shown in Fig. 4C and D, the majority of *dnaA* cells showed either no detectable signal (63%) or very low levels of fluorescence (33%), in line with our previous data showing that synthesis has mostly ceased in *dnaA* cells 90 min after the shift to the restrictive temperature (17, 23) (see Fig. S2A in the supplemental material). In contrast, 75% of *dnaA rnhA* cells showed robust levels of signal distributed over the entire length of the cell (Fig. 4C and D), suggesting robust levels of EdU incorporation even in the absence of *oriC* firing, in line with our fluorescence microscopy data (Fig. 4B), cell growth (Fig. 4A), and BrdU incorporation data (see Fig. S2A). Some of these cells showed multiple “spots” of synthesis, which is likely to be explained by multiple replisomes generating multiple stretches of newly synthesized DNA (Fig. 4D). Only 4% of cells showed no signal. *dnaA recG* cells, however, showed a mixed population (Fig. 4C and D). The majority of cells showed either no fluorescence (44%) or a low signal (27%) (Fig. 4D). Thus, 71% of cells showed fluorescence levels comparable to the levels observed in a *dnaA* single mutant, in line with the observed 75% of cells showing either a single origin and terminus focus or two segregated chromosomal copies (Fig. 4B). It is tempting to speculate that the BrdU incorporation observed in chromosomal areas away from the termination zone stems only from a fraction of cells. Indeed, 29% of cells showed a fluorescence signal in either several distinct locations, likely caused by multiple replisomes, or distributed over the entire length of the cell (Fig. 4D), implying high levels of synthesis. These high levels of synthesis will contribute rather significantly to the BrdU signal observed outside the termination area (see Fig. S2A). We cannot exclude that some synthesis takes place away from the termination area in all *recG* cells. However, if this is the case the levels of synthesis must be so low that they cannot be detected in our EdU pulse-labeling assay, in line with the lack of growth observed (Fig. 4A).

### Cells lacking RNase HI deal with genotoxic damage similar to wild-type cells.

**FIG 5:**
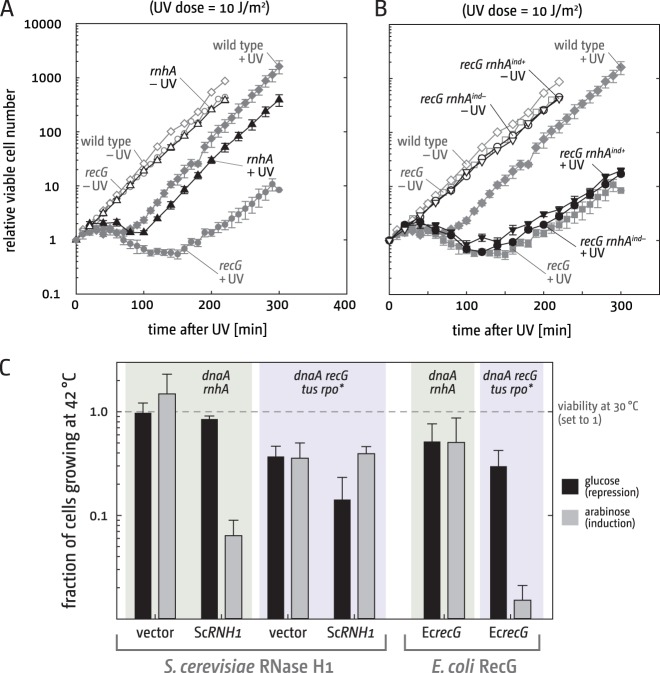
Complementation of *rnhA* and *recG* cells with *E. coli* RNase HI and yeast RNase H1. (A) Viable cell replication following irradiation (see Materials and Methods). The strain used was N4704 (*rnhA*). Data for the irradiated cells are means (±standard error [SE]) from three experiments. The data for the unirradiated cells are means from two experiments that gave almost identical values. Data for MG1655 and the *recG* strain were replotted from reference 15 for comparison. All experiments were performed under comparable conditions with identical equipment. (B) Filamentation of UV-irradiated *recG* cells with increased levels of *rnhA* expression. The strain used was AM2304 (*recG proB*::*rnhA^ind^*). This strain has, in addition to the functional *rnhA* gene, a second *rnhA* copy under control of the p*araBAD* promoter integrated into the *proB* gene (33). Growth in the presence of arabinose will induce additional expression of RNase HI, whereas glucose will repress expression, resulting in native expression levels. Data for irradiated cells are means ± SE from three experiments. Data for unirradiated cells are means from two experiments with almost identical values. Data for MG1655 (wild type) and N4560 (*recG*) were replotted from reference 15 for comparison. Experiments were performed under comparable conditions. (C) Origin-independent synthesis in *dnaA rnhA* cells and dnaA recG tus rpo* cells expressing either *Saccharomyces cerevisiae* *RNH1* (pECR22 [labeled “Sc*RNH1*”]) or *E. coli* *recG* (pDIM104 [labeled “Ec*recG*”]) gene. Expression is either repressed by 0.2% glucose or stimulated by 0.05% arabinose, as indicated. All numbers represent the fraction of colonies relative to the 30°C control, which was set to 1 (dashed line). Data are means from at least 3 independent experiments (±standard deviation [SD]). Growth of the *dnaA rnhA* vector control is stronger in medium with arabinose, which most likely reflects the broth sensitivity of *dnaA rnhA* cells. The strains used were RCe552 (*dnaA rnhA* pECR22 [RCe557 for vector control]), SLM1008 (dnaA recG tus rpo* pECR22 [SLM1010 for vector control]), SLM1104 (*dnaA rnhA* pDIM104), and RCe326 (dnaA recG tus rpo* pDIM104).

We demonstrated before that genotoxic stress triggers dramatic chromosomal overreplication, a filamentation phenotype, and severe segregation defects in *recG* cells (15, 17). To test what happens when damage-induced synthesis is triggered in cells lacking RNase HI, we determined the filamentation period after UV exposure. As shown in Fig. 5A, *rnhA* cells show a minor elongation of the filamentation period following UV exposure. However, this effect is moderate, and time-lapse microscopy confirmed that the filaments formed break down into normal-sized cells (see Fig. S3 in the supplemental material), in contrast to *recG* cells (15, 17). In addition, *dnaA rnhA* cells show comparable levels of synthesis before and after UV irradiation (see Fig. S2A in the supplemental material), and an excessive accumulation of chromosomal areas is not observed (see Fig. S2B), highlighting that *rnhA* cells cope with UV-induced synthesis and the resulting increase in fork collision events similar to wild-type cells.

### Initiation of synthesis at R-loops.

The fact that both RecG and RNase HI can process R-loops *in vitro* (11–13) has led to the idea that origin-independent synthesis in *rnhA* and *recG* cells might be initiated at R-loops (8, 14). To investigate whether R-loops are responsible for some of the phenotypes of *recG* cells, we tested whether increased levels of native *E. coli* RNase HI can suppress the extended filamentation period in UV-irradiated *recG* cells (15). *E. coli* *rnhA* cannot be expressed at high levels from a multicopy plasmid, as this results in cellular toxicity (33). We therefore used a strain in which a second functional copy of the *E. coli* *rnhA* gene under control of the p*araBAD* arabinose-controlled promoter was integrated into the chromosome (33). This allowed us to compare the filamentation period in UV-irradiated *recG* cells with native (promoter repressed by glucose) or increased (arabinose-induced) levels of RNase HI. As shown in Fig. 5B, increased levels of RNase HI had no effect on the filamentation period of cells lacking RecG.

Furthermore, we investigated whether the expression of *Saccharomyces cerevisiae* RNase H1 (encoded by *RNH1*), which hydrolyzes the RNA from RNA:DNA hybrids (34), can suppress origin-independent synthesis in *rnhA* and *recG* cells. It was shown before that some of the phenotypes of *rnhA* cells can be complemented by the expression of yeast RNase H from a high-copy-number plasmid (35). We cloned the *RNH1* gene into pBAD24 to allow arabinose-controlled transcription. Expression of yeast *RNH1* indeed reduced growth of *dnaA rnhA* cells at 42°C over 20-fold (Fig. 5C). In contrast, it did not show any suppression of growth of dnaA recG tus rpo* cells at 42°C (Fig. 5C). Thus, neither the expression of the yeast *RNH1* gene nor the overexpression of *E. coli* RNase HI shows any effect on the phenotype of *recG* cells, suggesting that R-loops are either not accessible to RNase HI or are not responsible for the phenotypes of *recG* cells. In line with this, expression of *E. coli* *recG* reduced the ability of dnaA recG tus rpo* cells to grow at 42°C 20-fold, while growth of *dnaA rnhA* cells at 42°C was unaffected (Fig. 5C).

### Head-on collisions of replication and transcription severely impede cell growth.

**FIG 6:**
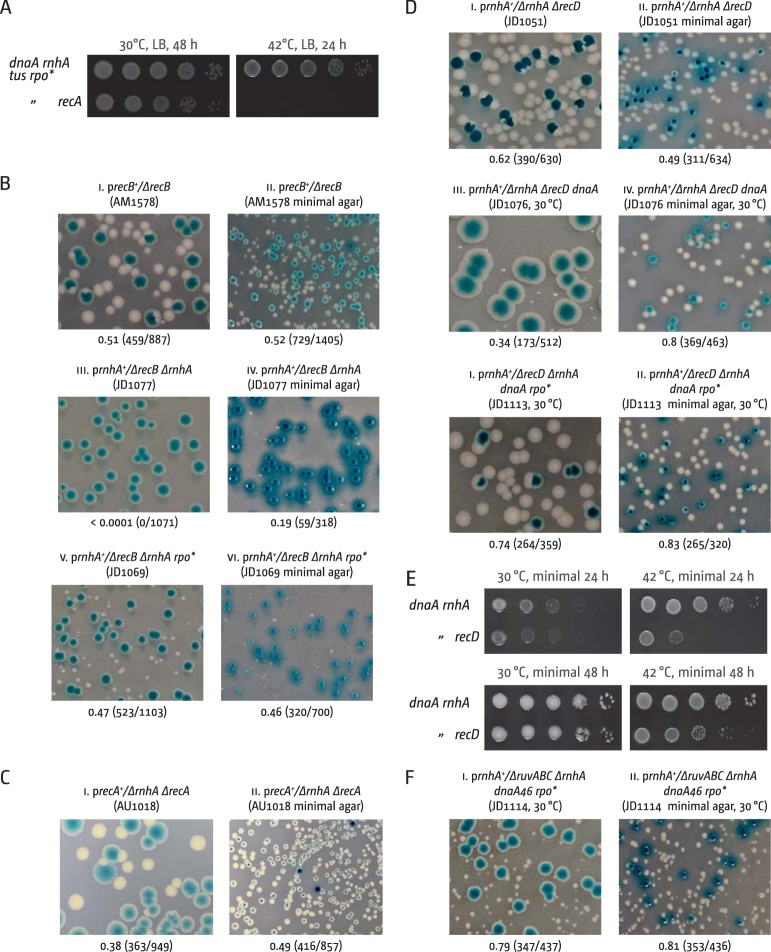
Effect of an *rnhA* deletion in recombination-deficient backgrounds. (A) Spot dilution assay to evaluate origin-independent growth in dnaA rnhA tus rpo* recA cells. The strains used were RCe309 (dnaA rnhA tus rpo*) and RCe529 (dnaA rnhA tus rpo* recA). (B to D) Maintenance of viability of *rnhA* cells in the absence of RecA, RecB, and RecD. The plate photographs shown are of synthetic lethality assays, as described in Text S1 in the supplemental material. The relevant genotype of the construct used is shown above each photograph, with the strain number in parentheses. The fraction of white colonies is shown below with the number of white colonies/total colonies analyzed in parentheses. Note that for panel Diii, an incubation time longer than usual was used (80 h for LB) to visualize the existing but very small white colonies. The plasmids used are pAM375 (*recB^+^*), pAM383 (*recA^+^*), and pAM490 (*rnhA^+^*) (see Text S1). (E) Spot dilution assays to evaluate origin-independent growth in *dnaA rnhA* cells in the absence of RecD. The strains used were AU1066 (*dnaA46 rnhA*) and JD1081 (*dnaA rnhA recD*). (F) Suppression of the synthetically lethal interactions of *rnhA* with *ruvABC* by rpo*. The plasmid used was pAM490 (*rnhA^+^*) (see Text S1).

Overreplication in *recG* cells depends critically on RecA recombinase, as was shown before for *rnhA* cells (8). We confirmed this for our dnaA rnhA tus rpo* background (Fig. 6A). Furthermore, overreplication in *recG* cells required the recombinase activity of RecBCD (*recB*) but not the ExoV activity (*recD*) (10), and growth of dnaA recG tus rpo* cells was completely abolished in the absence of the RuvABC Holliday junction resolvase (10). We wanted to perform a similar investigation in *dnaA rnhA* cells, which proved surprisingly difficult and required us to use a synthetic lethality assay. This assay employs the unstable pRC7 plasmid, which is rapidly lost, with cloned genes of interest to cover for chromosomal deletions (36, 37). pRC7 carries a functional *lac* operon, and its loss can be revealed in a *lac* mutant background on plates containing the β-galactosidase indicator X-Gal (5-bromo-4-chloro-3-indolyl-β-d-galactopyranoside) by the formation of white colonies or white sectors within blue (*lac^+^*) colonies, depending on whether plasmid loss occurred before or after plating (Fig. 6Bi).

Synthetic lethality of *rnhA recB* cells was reported before (38), and we confirmed that *rnhA recB* cells were unable to grow without an *rnhA^+^* covering plasmid, resulting in the formation of only blue colonies (Fig. 6Biii). In contrast, *rnhA recA* cells proved viable (Fig. 6C). Thus, cells lacking RNase HI rely on RecB, but not RecA, for survival, much in contrast to *recG recB* cells, which are viable (39). Some white colonies were observed on minimal medium (Fig. 6Biv), but these were very small, indicating a strong growth defect. Origin-independent synthesis was shown to continue in *rnhA*(Ts) *recB* cells upon shift to 42°C in the absence of origin firing (40), suggesting that origin-independent synthesis is initiated, but forks have problems progressing. The fact that the broth sensitivity of *dnaA rnhA* cells at 42°C is suppressed by an rpo* point mutation indicates that origin-independent synthesis in *rnhA* cells triggers replication-transcription conflicts. Indeed, we found that rnhA recB rpo* cells, even though exhibiting a severe growth defect, are viable without a covering plasmid (Fig. 6Bv), in line with the idea that RecBCD is vitally important to process intermediates resulting from replication-transcription encounters, as was shown for both *E. coli* and *Bacillus subtilis* (41, 42). As rnhA recB rpo* cells are viable, we attempted to generate a dnaA rnhA recB rpo* construct to investigate growth at 42°C. However, the sickness of rnhA recB rpo* cells led to the rapid accumulation of suppressor mutations, which made generation of this construct impossible.

We had no difficulty in generating *rnhA recD* cells (Fig. 6D). However, when we tried to generate a *dnaA rnhA recD* construct, we found cells to be extremely sick on LB broth at the permissive temperature of 30°C (Fig. 6Diii). It appears that the ExoV activity of RecBCD is dispensable in *rnhA* cells but becomes vital, even at permissive temperature, if a *dnaA*(Ts) allele is introduced, suggesting that the *dnaA46* allele might have a defect even at permissive temperature, as reported previously (43, 44). In line with this hypothesis, we noted that a *dnaA rnhA* construct in which the *dnaA46* allele is covered by a wild-type copy of *dnaA* on the covering plasmid showed noticeably smaller white colonies at 30°C on LB but not on minimal medium (cf. Fig. S4Ai and S4Aii in the supplemental material), while *dnaA recG* cells show no such difference (see Fig. S4Aiii and S4Aiv). Thus, it appears that *rnhA recD* cells are viable as long as *oriC* activity is unperturbed. As an rpo* mutation greatly improved the viability of *dnaA rnhA recD* cells on LB broth at 30°C (Fig. 6D), it appears that replication-transcription encounters are responsible for the problems observed.

As *dnaA rnhA recD* cells are viable on minimal medium (Fig. 6Div), we were able to investigate growth at 42°C. The absence of RecD slowed growth of *dnaA rnhA* cells, as indicated by the slow colony formation (Fig. 6E; see Fig. S4B in the supplemental material), much in contrast to *recG* cells, where quicker growth was observed (10). However, colony numbers of *dnaA rnhA recD* cells at 42°C were only mildly reduced, supporting the idea that origin-independent synthesis in *rnhA* cells is able to continue in the absence of RecD, as suggested (8).

Generation of a *dnaA rnhA ruvABC* background also proved difficult. Surprisingly, even in the presence of a *ruvABC^+^* covering plasmid, *dnaA rnhA ruvABC* cells grew very slowly (see Fig. S4Av in the supplemental material). The total absence of white colonies suggests that *dnaA rnhA ruvABC* cells are synthetically lethal. In contrast to the robust viability of *rnhA recD* cells (Fig. 6D), we reported previously that *rnhA ruvABC* cells show a growth defect (18). As *dnaA rnhA* cells also showed a growth defect, the extremely slow growth of *dnaA rnhA ruvABC* p*ruvABC^+^* cells is likely to be due to multiple harmful genetic interactions. As for *recB* and *recD*, it was reported that a deletion of *ruvABC* did not affect initiation of origin-independent synthesis in *rnhA* cells (8). Thus, Holliday junction resolution appears to be important in cells lacking RNase HI, especially so if *oriC* firing is impeded, and it seems to be important for the processing of replication forks, rather than being required for the initiation of origin-independent synthesis. In line with this hypothesis, we found that an rpo* point mutation improved the viability of *dnaA rnhA ruvABC* cells enough to allow us to generate a dnaA rnhA ruvABC rpo* p*rnhA* construct, which showed small but clearly visible white colonies on LB and larger white colonies on minimal medium (Fig. 6F). Taken together, our results support the idea that the problems observed in the absence of RecB, RecD, and RuvABC are induced by replication-transcription conflicts, as multiple replication forks are traversing the chromosome in a direction opposite to normal.

## DISCUSSION

**FIG 7:**
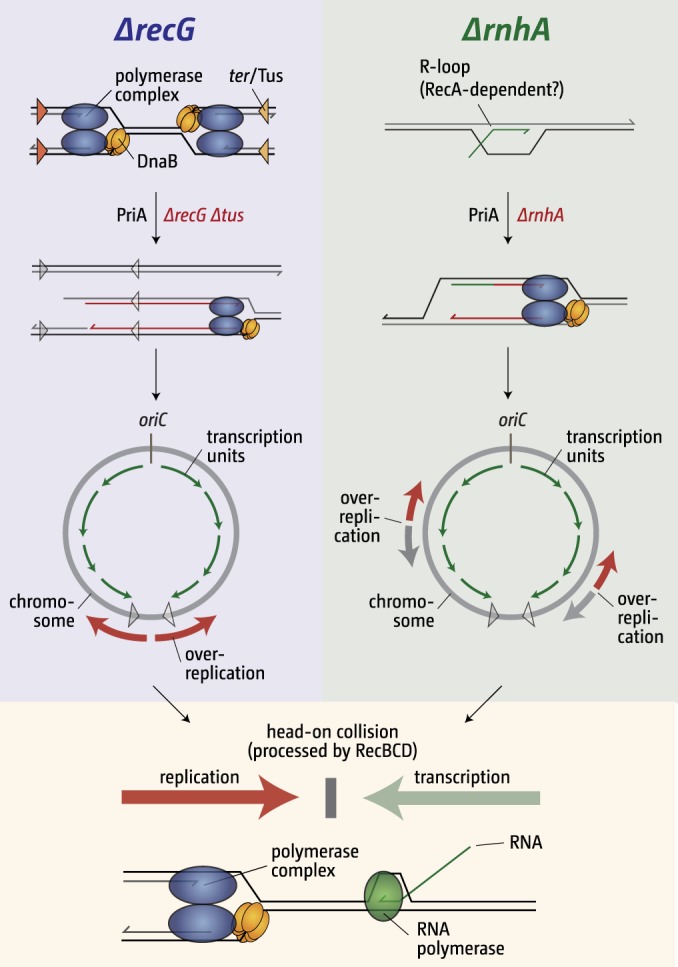
Overreplication in cells lacking RNase HI and RecG is triggered by different events but has similar consequences. See the text for details.

Cells lacking either RNase HI or RecG show substantial levels of origin-independent replication of the chromosome, a form of replication that is independent of the DnaA initiator protein and which was originally described by Kogoma and coworkers as stable DNA replication (SDR) (8). Since both RNase HI and RecG eliminate R-loops *in vitro*, the former by degrading the RNA (11), the latter by unwinding RNA:DNA hybrids (12, 13), it was assumed that an accumulation of R-loops might be the trigger for SDR in both cases (8, 14). However, the data presented in this study demonstrate that this is not so. They also reveal that SDR increases conflicts between replication and transcription, thereby threating the stability of the genome and cell viability (Fig. 7).

We found that, whereas cells lacking RecG show a pronounced initiation of SDR at a single location in the termination area (10), cells lacking RNase HI show initiation at multiple sites around the chromosome (Fig. 1), as reported previously (9, 24). One of these is located in the termination area, but the effect of chromosome linearization on synthesis in this region (Fig. 3) suggests that the mechanism responsible for this synthesis is different from that operating in the absence of RecG.

This idea is further supported by our PriA results. Although initiation of SDR depends in both cases on the ability of PriA helicase to load the replicative helicase, DnaB, at a branched DNA substrate and thereby to prime replisome assembly, we demonstrated that the substrates exploited for this purpose are different. In a previous study, it was shown that the ability of SDR to promote growth of dnaA recG tus rpo* cells at 42°C specifically requires the ability of PriA to unwind a replication fork with a lagging strand only, the equivalent of a 3′ flap (10). It was suggested that 3′ flaps are generated when replication forks fuse in the terminus area and that these flaps accumulate in the absence of RecG, providing a suitable template for DnaB loading. We found that this particular activity of PriA is dispensable for the initiation of SDR in dnaA rnhA tus rpo* cells (Fig. 2). Further confirmation that PriA exploits different substrates to initiate SDR in *rnhA* and *recG* cells came when we found that yeast *RNH1* reduces growth of *dnaA rnhA* cells at 42°C 20-fold, whereas it does not reduce the viability of dnaA recG tus rpo* cells at all (Fig. 5).

These data, when taken together with studies revealing that recombination mutations inactivating RecA or eliminating components of RecBCD or RuvABC have contrasting effects on the viability of *rnhA* and *recG* cells (Fig. 6) (8, 10, 40), reinforce the idea that the SDR detected in these cells is the result of very different events: the accumulation of R-loops in the former as opposed to persisting 3′ flaps in the latter (Fig. 7).

However, regardless of this difference, our studies reveal that the SDR induced in both cases has one common feature. By compromising the normal replichore arrangement, SDR leads to increased conflicts between replication and transcription (Fig. 7). This is highlighted by the impact of an rpo* mutation that destabilizes ternary transcription complexes. It strongly improves the viability of *dnaA rnhA* and *dnaA recG* cells at 42°C (Fig. 1D) (10), although in the latter case, the effect depends on the deletion of *tus*. This is hardly surprising, as SDR in *recG* cells is initiated within the replication fork trap in the terminus region, thereby preventing forks from proceeding toward *oriC* in the presence of functional *ter*/Tus complexes. Multiple initiation sites in *rnhA* cells avoid this difficulty. Replication-transcription collisions are severe enough to threaten the viability of *rnhA* cells in the absence of factors such as RecBCD (Fig. 6). RecBCD was shown before to be vital for the processing of forks stalled at transcription complexes (41, 42, 45). Recent data from *B. subtilis* suggest that restart at replication-transcription conflict sites is dependent on RecA-driven D-loop formation initiated either by RecBCD homologues or RecFOR (42). While *rnhA recB* cells are synthetically lethal (Fig. 6), both *rnhA recA* (Fig. 6) and *rnhA recF* (data not shown) double mutants could be constructed without difficulty, highlighting perhaps the importance of RecBCD for replication restart at replication-transcription conflict sites in *E. coli*, as suggested (41). RecBCD is likely to be required for processing replication-transcription conflicts in *recG* cells, but as overreplication in the termination area in *recG* cells is strictly dependent on RecB recombinase activity (10), replication-transcription conflicts simply do not arise in *recG recB* double mutants, explaining why they are viable.

Our results indicate that replication-transcription conflicts are a major problem for cells lacking RNase HI, an effect exacerbated when DnaA activity is compromised (Fig. 1 and 6; see Fig. S4 in the supplemental material). The broth sensitivity of *dnaA rnhA* cells was observed in an earlier study but never explained (8). Our finding that an rpo* mutation suppresses this effect (Fig. 1) makes it quite clear that it is most likely the result of replication-transcription conflicts.

In *B. subtilis*, *rrn* operons have been identified as a severe impediment to DNA replication. The high levels of transcription can interfere with replication even if both processes are proceeding codirectionally (46), a result that might also be true for *E. coli* (47). Head-on encounters are thought to be even more deleterious than codirectional encounters. Indeed, the most dramatic effect on replication in *B. subtilis* cells in which a part of the chromosome is replicated opposite to normal is observed at *rrn* operons, an effect much alleviated in minimal salts medium (48). A comparison of our *rnhA* and *dnaA rnhA* replication profiles reveals a significant “step” that coincides with the location of the *rrnCABE* operon cluster (3.94 to 4.21 Mbp) (see Fig. S5 in the supplemental material), a step that is much reduced in dnaA rnhA tus rpo* cells (cf. Fig. 1C and F; see Fig. S5). This observed “step” indicates a slowing of replication forks in this area, in line with head-on collisions between replication and transcription as forks coming from the initiation site at 4.54 Mbp will progress into this area in the wrong orientation. Indeed, we observed recently that replication forks initiated at an ectopic replication origin are significantly impeded at the *rrn* operons (47), as reported for *Bacillus subtilis* (48, 49). If replication forks are slowed or blocked at *rrn* operons, this would also explain the very low marker frequency at the location of *oriC* in *dnaA rnhA* cells. As origin-independent synthesis is initiated between 4.54 and 0.4 Mbp, which is relatively close to *oriC*, a higher marker frequency level might be expected unless synthesis traversing toward *oriC* is blocked at *rrnCABE.*

To conclude, our data from this as well as our previous studies (10, 47) provide strong additional *in vivo* evidence that codirectionality of transcription and replication and their interaction is likely to be a particularly important driving force that has shaped the chromosomal architecture in bacteria. Regardless of the mechanism of initiation, replisomes traversing the chromosome in the wrong orientation induce collision events with transcription, which require subsequent processing by recombination proteins (Fig. 7), with RecB being of particular importance, as reported previously (41, 42). The threat to the integrity of the genome is likely to be substantial. However, our data also support the idea that fork fusion events are another important factor that has shaped the chromosomal architecture (Fig. 7) (10, 47, 50). The importance of dealing with fork fusion intermediates is highlighted by the multiple proteins involved in their processing, such as RecG, 3′ exonucleases, polymerase I, and RecBCD (10, 18, 28, 51). It is significant that *recG rnhA* is synthetically lethal (8, 52), and it remains to be established whether this synthetic lethality might be caused by the consequences of too many unprocessed replication fork fusions, replication-transcription conflicts, or both.

## MATERIALS AND METHODS

### Bacterial strains and general methods.

The *Escherichia coli* K-12 strains used are derivatives of MG1655 (see Table S1 in the supplemental material). Strains were constructed via P1*vir* transductions (53) or single-step gene disruptions (54). For the compositions of LB broth and 56/2 minimal medium, see Text S1 in the supplemental material. The *dnaA46* allele encodes a thermosensitive DnaA protein that is inactive at 42°C. For assessment of growth without DnaA initiation, cultures of *dnaA46* constructs grown at 30°C to an *A*_600_ of 0.4 were diluted in 10-fold steps to 10^−5^ before spotting of 10-µl samples of each dilution on LB agar. Duplicate plates were incubated at 30°C and 42°C. Chromosome linearization was conducted as described previously (31). The synthetic lethality assay, fluorescence microscopy, BrdU labeling and detection via immunostaining, the determination of the multiplication of cells surviving UV irradiation, and the generation of a loess regression curve have been described before and are detailed in the supplemental material.

### Multiplication of *dnaA* cells following a shift to the restrictive temperature.

To quantify the number of cell divisions of *dnaA46* strains following the shift to 42°C, strains were grown with vigorous aeration in LB broth to an *A*_600_ of 0.2 at 30°C. The samples were transferred into a water bath prewarmed at 42°C. Samples were removed as indicated and diluted in conditioned medium, which was created by growing the wild-type strain in fresh LB broth to an *A*_600_ of 0.2 with subsequent sterile filtration. Samples were mixed with 2.5 ml of molten 0.6% top agar kept at 42°C and plated on LB agar. Colonies were counted after incubation for 48 h at 30°C.

### Suppression of the *rnhA* phenotype by yeast RNH1.

For details of the generation of the yeast *RNH1* expression plasmid pECR22, see Text S1 in the supplemental material. Cells were grown in LB broth supplemented with ampicillin (50 µg/ml) to an *A_600_* of 0.48 at permissive temperature (30°C). Samples were diluted in 10-fold steps from 10^−1^ to 10^−5^ before each dilution was spotted onto minimal medium supplemented with either 0.2% glucose or 0.05% arabinose. Two identical plates were generated, and one was incubated at 30°C, while the other was incubated at 42°C. Colonies were counted, and the viable titer at 30°C was set to 1. Titers at 42°C on medium containing either glucose or arabinose were calculated as a fraction of the viable titer measured at 30°C.

### Marker frequency analysis by deep sequencing.

Samples from cultures of a strain grown overnight in LB broth were diluted 100-fold in fresh broth and incubated at 37°C with vigorous aeration until an *A*_600_ reached 0.4. The culture was then diluted again 100-fold in prewarmed fresh broth and grown until an *A*_600_ of 0.4 was reached. Samples were flash-frozen in liquid nitrogen at this point for subsequent DNA extraction. Strains harboring the *dnaA46* allele were grown overnight at 30°C. Upon dilution, cells were incubated for 60 min at 30°C and then shifted to 42°C for the remainder of the experiment. For exponentially growing wild-type cells, a culture was grown in parallel under the same conditions. For a stationary-phase sample, a wild-type culture was grown overnight until the culture had saturated and showed no further increase in the *A*_600_. DNA was extracted using the GenElute bacterial genomic DNA kit (Sigma-Aldrich). Marker frequency analysis was performed using Illumina HiSeq 2000 sequencing (fast run) to measure sequence copy number. Enrichment of uniquely mapping sequence tags, in 1-kb windows, was calculated for an exponentially growing (replicating) sample relative to a nonreplicating stationary-phase wild-type sample to correct for differences in read depth across the genome and to allow presentation of the data as a marker frequency, as described previously (9, 19, 20).

### EdU click labeling of newly replicated DNA in *E. coli.*

EdU click labeling of newly replicated DNA in *E. coli* was performed essentially as described previously (32), using the Click-iT Plus EdU Alexa Fluor 488 kit from Life Technologies. Briefly, *dnaA46* cells and derivatives were grown at 30°C to an *A*_600_ of 0.2 before shift to 42°C for 90 min to achieve a run-out of ongoing synthesis. EdU was added (30 µg/ml final concentration), and cells were labeled for 15 min; 2-ml sample aliquots were fixed by adding 13 ml of 90% methanol. The components of the click labeling reaction mixture were prepared according to the manufacturer’s description. The labeling reaction was performed as described previously (32).

### Imaging flow cytometry.

Imaging flow cytometry was conducted by using the Imagestream^X^ Mark II system (Amnis, Inc., Seattle, WA). This permits image capture of single cells in flow using a maximum of six optical channels. Images of between 6,000 and 12,000 cells were acquired at a 60× magnification for bright-field (BF) microcopy and for Alexa Fluor 488, using the 488-nm excitation laser set to 50 mW. The side scatter was established with a 785-nm laser set to 2 mW. Fluorescence levels per background were analyzed with the IDEAS imaging flow cytometry software V6.1. Following gating to identify single and in-focus cells, fluorescence levels were compared to images of the relevant cells, identifying cells with no detectable fluorescence, low fluorescence levels, or high signal intensity, as presented.

## SUPPLEMENTAL MATERIAL

Text S1  Supplemental methods. Download Text S1, DOCX file, 0.04 MB

Text S2  Supplemental references. Download Text S2, DOCX file, 0.02 MB

Figure S1  Verification of chromosome linearization in *dnaA rnhA tos* N15 lysogen cells. (A) Schematic representation of the area around *dif* with and without integrated *tos-kan* site. The linearization verification primers are shown in green (for primer sequences, see reference 12), and the PCR product sizes in wild-type cells and integrants are indicated. (B) PCR products generated with the linearization verification primers for *tos-kan* cells lysogenized with phage N15 (RCe403 [lane 1]), *tos-kan* cells (RCe401 [lane 2]), and an N15 lysogen of *dnaA rnhA* cells without the *tos-kan* linearization site (RCe383 [lane 3]). The shift of the PCR product size in a nonlinearized strain as shown in lane 2 indicates the presence of the *tos-kan* cassette. Linearization of the chromosome (lane 1) prevents formation of a specific PCR product since the chromosome is cleaved between the primer binding sites. The absence of a detectable PCR product confirms that the amount of circular chromosomes unprocessed by TelN in the population is very low, as reported previously (12). (C to F) Verification of chromosome linearization by pulsed-field gel electrophoresis (PFGE). If the *tos* site is cleaved by TelN, an additional band becomes visible on PFGE gels. The *tos* site is located in the 273.6-kb NotI fragment between positions 1337601 and 1611219 (C [highlighted in green]), and cleavage by TelN splits it into two fragments, one of which is 251.2 kb and the other of which is 22.4 kb (E and F [highlighted in green]). The 251.2-kb fragment moves into the quadruplet around 250 kb and thus is hidden in between other fragments (E). The smaller 22.4-kb fragment, however, becomes visible as an additional fragment at the bottom of the gel highlighted by a black arrow (D and E). A negative image is shown for clarity. Chromosomal DNA was prepared from RCe607 (*rnhA* N15 lysogen), RCe605 (*rnhA tos-kan*), and RCe608 (*rnhA tos-kan* N15 lysogen). Download Figure S1, PDF file, 0.3 MB

Figure S2  Damage-induced synthesis in cells lacking RNase HI. (A) Fluorograph showing a side-by-side comparison of BrdU incorporation into the chromosome of irradiated and mock-irradiated *dnaA rnhA* cells (AU1066). A schematic NotI restriction pattern of the *E. coli* chromosome is shown on the left, indicating the distance from *oriC* to each end of the shown fragments. Fragments clockwise and anticlockwise of *oriC* are shown in red and blue, respectively. Data for irradiated and mock-irradiated *dnaA* (AU1054) and *dnaA recG* (AU1091) cells were reproduced from reference 9 for comparison. The experiments were performed under comparable conditions on the same equipment. (B) Fluorescence microscopy showing replication of origin (red foci) and terminus (green foci) areas of the chromosome (combined phase-contrast and fluorescence images are shown) following the shift to the restrictive temperature in UV-irradiated cells. The strains used were AU1091 (*dnaA recG*) and AU1066 (*dnaA rnhA*). The incubation time after irradiation is indicated. Download Figure S2, PDF file, 1.6 MB

Figure S3  Effect of RNase HI on cell cycle progression of UV-irradiated cells. (A to C) Time-lapse photography following growth of single cells after UV irradiation. The strain used was N4704 (*rnhA*). Images of MG1655 (wild type) and N4560 (*recG*) were reproduced from reference 7 for comparison. White arrows indicate last divisions after irradiation before cells start to filament. Dark arrows illustrate dead cells budded off *recG* and *rnhA* filaments either showing no further divisions or bursting, leaving a “ghost.” While there is some extended filamentation in *rnhA* cells, the later time points clearly show that the filaments formed break up into small and normally growing cells. Experiments were performed under comparable conditions with the same equipment. Download Figure S3, PDF file, 1.4 MB

Figure S4  Effect of *recD* and *ruvABC* on cell survival and growth of cells lacking RNase HI. (A) Maintenance of cell viability in *dnaA rnhA* and *dnaA rnhA ruv* cells. The plasmids used were pAU101 (*dnaA^+^*) and pAM390 (*ruvABC^+^*) (for plasmids, see Text S1 in the supplemental material). Panels 1 to 4 show plate photographs of synthetic lethality assays, as described in Materials and Methods. The relevant genotype of the construct used is shown above each photograph, with the strain number in parentheses. The fraction of white colonies is shown below with the number of white colonies/total colonies analyzed in parentheses. For panel v, a transductant of a p*ruvABC*^+^/*ruvABC* *rnhA* *dnaA* cross was streaked to single colonies on plates containing X-Gal/IPTG without ampicillin. (B) Spot dilution assays to evaluate origin-independent growth in *dnaA rnhA* cells in the absence of RecD. The strains used were AU1066 (*dnaA rnhA*) and JD1081 (*dnaA rnhA recD*). Download Figure S4, PDF file, 0.7 MB

Figure S5  Overlay of replication profiles of *rnhA* derivatives. (A) Comparison of the replication profiles of *dnaA rnhA* and dnaA rnhA tus rpo* cells. Introduction of an rpo* point mutation changes the “step” observed at the position of the *rrnCABE* operon cluster, as indicated by dotted lines. The data sets are reproduced from Fig. 1. (B) Comparison of the replication profiles of *rnhA* and *dnaA rnhA* cells. The data sets are reproduced from Fig. 1. Download Figure S5, PDF file, 0.4 MB

Table S1  List of all *Escherichia coli* K-12 constructs used in this study. Table S1, DOCX file, 0.05 MB
