# Supplementary material for: The Consequences of Replicating in the Wrong Orientation: Bacterial Chromosome Duplication without an Active Replication Origin
Source: mBio. 2015 Nov 3;6(6):e01294-15. doi: 10.1128/mBio.01294-15 (PMC4631800; doi:10.1128/mBio.01294-15)
Supplement: Text S1 — Supplemental methods. Download [file mbo005152518s1.docx]

# Supplementary Methods

## Growth media

Luria broth (LB) and agar was modified from (1) as follows: 1% tryptone (Bacto™, BD Biosciences), 0.5% yeast extract (Bacto™, BD Biosciences) and 0.05% NaCl (Sigma Aldrich). Glucose was omitted and the pH adjusted to 7.4. Minimal medium “56” was prepared according to (2): 74 mM KH_2_PO_4_, 120 mM Na_2_HPO_4_, 3.4 mM MgSO_4_ · 7 H_2_0, 30 mM (NH)_2_SO4, 85 µM Ca(NO_3_)_2_ and 3.6 µM FeSO_4_ · 7 H20. The pH was adjusted to 7.4. This was used at one-half strength, denoted 56/2. The term “minimal medium” refers to 56/2 containing 0.32% glucose.

## Plasmids used in this study

Plasmids pJJ100, pAM374, pAM375, pAM383, pAM390, pAM403 and pAM490 have been described elsewhere (3, 4). All carry *lac*^+^. In addition, pJJ100 carries *recG*^+^, pAM383 carries *recA^+^*, pAM390 carries *ruvAB^+^* and *ruvC^+^*, pAM403 carries *rep^+^* and pAM490 carries *rnhA*^+^. All genes include their native promoter regions. pAM374 and pAM375 carry *priA*^+^ and *recB*^+^, respectively, which are expressed from the *lac* promoter and require IPTG for expression. pAU101 is a derivative of pRC7 carrying the coding sequence for *dnaA^+^* including its native promoter, which was PCR amplified from MG1655 using 5’ and 3’ primers incorporating *Apa*I sites. The PCR product was cloned into the *Apa*I site within *lacI^q^* to give pAU101. The coding sequence inserted is transcribed in the same orientation as the disrupted *lacI^q^* gene. pAU101 complements the temperature sensitivity of the *dnaA46* strain AU1054. For generation of pECR22, *RNH1* was amplified from *Saccharomyces cerevisiae* chromosomal DNA with primers introducing restriction sites for *EcoRI* and *NheI* and cloned into pBAD24 under control of the arabinose promoter (5). pLau17 was used as a vector control (6). pDIM104, which carries the *recG* coding sequence cloned behind the p*araBAD* promoter of pBAD24, was described before (7).

## Single cell analyses

Cells were grown in LB broth to an *A_600_* of 0.2. Microscope slides were equipped with a Gene Frame® (ABgene) and filled with LB agar (0.8%). 2.5 µl of the culture was added on top, the samples irradiated, the gene frames sealed with a cover slip and the slides examined on a heated (sample temperature ~35°C) microscope stage (INSTEC, Inc.) with a BX-52 Olympus microscope equipped with a coolSNAP™HQ camera (Photometrics). Pictures were taken at 5 min intervals. Images were taken and analysed by MetaMorph 6.2 (Universal Imaging) and processed using MetaMorph and Adobe Photoshop CS6.

## Fluorescence Microscopy

Fluorescence microscopy was as described (8). Briefly, cells were grown to an *A_600_* of 0.2 in LB broth supplemented with 0.5 mM IPTG and 40 ng/ml anhydrotetracycline. A 1 ml sample was removed and expression induced by adding arabinose to 0.2%. The rest of the cells were pelleted, UV-irradiated on the surface of LB agar and resuspended in the original, but filter-sterilised, supernatant to continue incubation. 1 ml samples were removed every 30 min and expression induced with arabinose for 30 min. A small sample of cells was transferred to a thin 1% LB agarose layer on microscopic slides and visualized with a BX-52 Olympus microscope equipped with a coolSNAP™HQ camera (Photometrics). eCFP and eYFP foci were visualized using the JP4-CFP-YFP filterset 86002v2 (Chroma). Images were taken and analysed by MetaMorph 6.2 (Universal Imaging) and processed using MetaMorph and Adobe Photoshop CS6.

## 5-Bromo-2'-deoxyuridine (BrdU) labelling

BrdU labelling and detection via immunostaining was essentially as described (9). Cells were grown in 56/2 salts supplemented with 0.2% casamino acids and 0.32% glucose to an *A_600_* of 0.2. The culture was split into various 2 ml aliquots, 5-Bromo-2’-deoxyuridine (BrdU, Sigma) added to the first aliquot to 20 µg/ml and the cultures shifted to 42°C. At the times indicated BrdU was added to one of the remaining aliquots. The aliquots were labelled with BrdU for 8 min, pelleted and resuspended in 85 µl TEE buffer (10 mM Tris • HCl, 10 mM EGTA, 100 mM EDTA, pH 8.0), containing 0.05% lauroylsarcosine and 0.5% SDS. 85 µl of liquid 1.4% low melting point agarose was added and the mixture solidified in a disposable plug former (Bio-Rad) at 4°C. Plugs were treated with 10 mg/ml lysozyme in 3 ml TEE buffer containing 0.05% lauroylsarcosine and 0.5% SDS for 2 h at 37°C and then at 52°C overnight with 5 mg/ml proteinase K in 3 ml TEE containing 1% SDS. Plugs were washed in TEE for 30 min at 37°C, treated with 1 mM phenylmethane sulphonyl fluoride (freshly prepared as 100 mM stock solution in methanol) in fresh TEE for 1 h at 37°C, washed in fresh TEE for 30 min at 37°C and finally in 0.1 × TEE for 30 min at 37°C. The plugs were subsequently transferred into 300 µl restriction enzyme buffer and incubated for 30 min at room temperature, the buffer changed and 25 u of *Not*I (NEB) added. Chromosomal DNA was digested overnight and the fragments separated on a 0.8% agarose gel (Bio-Rad pulse field certified agarose) in 0.5 × TBE using a CHEF Mapper PFGE system (Bio-Rad), running with a gradient voltage of 6 V/cm, an included angle of 120°, and initial and final switch times of 1.65 and 32.45 sec, respectively, with a run time of 20 h at 14°C. DNA was transferred to a Hybond-N+ Membrane (GE Healthcare) by alkaline vacuum transfer and UV crosslinked (120 mJ/cm²). Blocking was achieved with TBS Tween (50 mM Tris • HCl, 150 mM NaCl, pH 8.0, 0.5% Tween 20) containing milk powder (2%). After blocking the membrane was incubated for 2 h in the presence of mouse anti-BrdU antibody (Santa Cruz), diluted 1:5000 in TBS Tween. Horse radish peroxidase conjugated secondary antibody (goat anti-mouse, Bio-Rad) was used at a dilution in TBS Tween of 1:10,000 for 1.5 h. The membrane was incubated with ECL Plus Western Blotting Detection Reagents (GE Healthcare) and the signal visualised either by exposure to X-Omat UV Plus film (Kodak) or by the ChemiDoc chemiluminescence detection system (Bio-Rad).

## Synthetic lethality assay

The synthetic lethality assay was performed as described (3, 10). In essence, a wild type gene of interest is cloned in pRC7, a *lac^+^* mini-F plasmid that is rapidly lost, and used to cover a null mutation in the chromosome, in a *lac^–^* background. A mutation in another gene of interest is then introduced into the chromosome. If the double mutant is viable, the plasmid-free cells segregated during culture will form *lac^–^* colonies on agar plates. If synthetically lethal, they will fail to grow and only *lac^+^* colonies formed by cells retaining the plasmid will be observed. When viability is reduced but not eliminated, the colonies formed by cells retaining the plasmid are noticeably larger than those formed by plasmid-free cells. To record the phenotype, cultures of strains carrying the relevant pRC7 derivatives were grown overnight in LB broth containing ampicillin to maintain plasmid selection, diluted 100-fold in LB broth and grown without ampicillin selection to an *A_600_* of 0.5 before spreading dilutions on LB agar or 56/2 glucose minimal salts agar supplemented with X-gal and IPTG. Plates were photographed and scored after 48 h (LB agar) or 72 h (56/2 agar) at 37°C. For growth at 30°C plates were incubated for 72 h (LB) or 96 h (56/2 agar).

## Multiplication of cells surviving UV irradiation

To monitor recovery of cells surviving UV-irradiation, strains were grown with vigorous aeration in LB broth to an *A_600_* of 0.2. The cells were pelleted, spread on the surface of a 9 cm LB agar plate to remove all UV-absorbing liquid and UV- or mock-irradiated. The surface was washed with the original filter-sterilised supernatant and diluted 10,000-fold in conditioned medium, which was created by growing the wild type strain in fresh LB broth to an *A_600_* of 0.2 with subsequent sterile filtration. The diluted cells were incubated in a 37°C shaking water bath and at each time point samples were removed, mixed with 2.5 ml of molten 0.6% top agar kept at 42°C and plated on LB agar. At later time points the samples were diluted a further 10- or 100-fold in conditioned medium before plating. Colonies were counted after incubation for 18–24h at 37°C.

## LOESS regression

A LOESS regression allows for a simplified visualisation of complex data sets. For a LOESS regression relatively simple models are fitted to defined small subsets of data points in order to develop a function describing the deterministic part of the variation in the data. Weighted least squares are used to fit a low-degree polynomial to a specified percentage of the data points. Data points are weighted by a smooth decreasing function of their distance to the smoothed point, giving more weight to points closer to the point whose response is being estimated, while less weight is given to points further away. We used a second order polynomial for local fit, tricube as weight function and set a fraction of data used for smoothing to 10%, which corresponds to a smoothing window around 460 kbp (11). To account for circularity of the chromosome, we used periodic boundary conditions.
