# Supplementary material for: The Consequences of Replicating in the Wrong Orientation: Bacterial Chromosome Duplication without an Active Replication Origin
Source: mBio. 2015 Nov 3;6(6):e01294-15. doi: 10.1128/mBio.01294-15 (PMC4631800; doi:10.1128/mBio.01294-15)
Supplement: Text S2 — Supplemental references. Download [file mbo005152518s2.docx]

## Supplementary References

1. **Luria SE, Burrous JW**. 1957. Hybridization between *Escherichia coli* and Shigella.
J Bacteriol **74**:461–476.

2. **Low B**. 1973. Rapid mapping of conditional and auxotrophic mutations in *Escherichia coli* K-12.
J Bacteriol **113**:798–812.

3. **Mahdi AA, Buckman C, Harris L, Lloyd RG**. 2006. Rep and PriA helicase activities prevent RecA from provoking unnecessary recombination during replication fork repair. Genes Dev **20**:2135–2147.

4. **Rudolph CJ, Upton AL, Briggs GS, Lloyd RG**. 2010. Is RecG a general guardian of the bacterial genome? DNA Repair **9**:210–223.

5. **Guzman LM, Belin D, Carson MJ, Beckwith J**. 1995. Tight regulation, modulation, and high-level expression by vectors containing the arabinose p*BAD* promoter. J Bacteriol **177**:4121–4130.

6. **Lau IF, Filipe SR, Søballe B, Økstad O-A, Barre F-X, Sherratt DJ**. 2003. Spatial and temporal organization of replicating *Escherichia coli* chromosomes. Mol Microbiol **49**:731–743.

7. **Rudolph CJ, Upton AL, Harris L, Lloyd RG**. 2009. Pathological replication in cells lacking RecG DNA translocase. Mol Microbiol **73**:352–366.

8. **Rudolph CJ, Upton AL, Lloyd RG**. 2007. Replication fork stalling and cell cycle arrest in UV-irradiated *Escherichia coli*. Genes Dev **21**:668–681.

9. **Rudolph CJ, Upton AL, Lloyd RG**. 2009. Replication fork collisions cause pathological chromosomal amplification in cells lacking RecG DNA translocase. Mol Microbiol **74**:940–955.

10. **Bernhardt TG, de Boer PAJ**. 2003. The *Escherichia coli* amidase AmiC is a periplasmic septal ring component exported via the twin-arginine transport pathway. Mol Microbiol **48**:1171–1182.

11. **Cleveland WS**. 1979. Robust Locally Weighted Regression and Smoothing Scatterplots.
J Am Stat Assoc **74**:829–836.

12. **Cui T, Moro-oka N, Ohsumi K, Kodama K, Ohshima T, Ogasawara N, Mori H, Wanner B, Niki H, Horiuchi T**. 2007. *Escherichia coli* with a linear genome. EMBO Rep **8**:181–187.

13. **Bidnenko V, Seigneur M, Penel-Colin M, Bouton MF, Dusko Ehrlich S, Michel B**. 1999. *sbcB sbcC* null mutations allow RecF-mediated repair of arrested replication forks in *rep recBC* mutants. Mol Microbiol **33**:846–857.

14. **Bachmann, B J**. 1996. Derivations and Genotypes of Some Mutant Derivatives of *Escherichia coli* K-12. *Escherichia coli* and Salmonella Cellular and Molecular Biology. Second Edition. ASM Press.

15. **Stockum A, Lloyd RG, Rudolph CJ**. 2012. On the viability of *Escherichia coli* cells lacking DNA topoisomerase I. BMC Microbiol **12**:26.

16. **Rudolph CJ, Upton AL, Stockum A, Nieduszynski CA, Lloyd RG**. 2013. Avoiding chromosome pathology when replication forks collide. Nature **500**:608–611.

17. **Rudolph CJ, Mahdi AA, Upton AL, Lloyd RG**. 2010. RecG protein and single-strand DNA exonucleases avoid cell lethality associated with PriA helicase activity in *Escherichia coli*.
Genetics **186**:473–492.

18. **Zhang J, Mahdi AA, Briggs GS, Lloyd RG**. 2010. Promoting and avoiding recombination: contrasting activities of the *Escherichia coli* RuvABC Holliday junction resolvase and RecG DNA translocase. Genetics **185**:23–37.

19. **Jaktaji RP, Lloyd RG**. 2003. PriA supports two distinct pathways for replication restart in UV-irradiated *Escherichia coli* cells. Mol Microbiol **47**:1091–1100.
