# Supplementary figures and images for: The Consequences of Replicating in the Wrong Orientation: Bacterial Chromosome Duplication without an Active Replication Origin
Source: mBio. 2015 Nov 3;6(6):e01294-15. doi: 10.1128/mBio.01294-15 (PMC4631800; doi:10.1128/mBio.01294-15)

**A** I. *dif* region in wild type *E. coli* cells

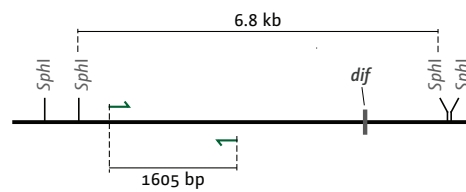

II. *dif* *tos-kan* construct

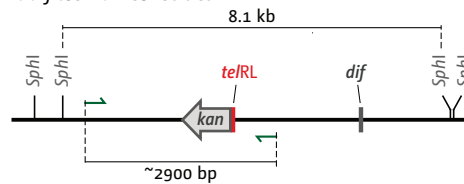

**B**

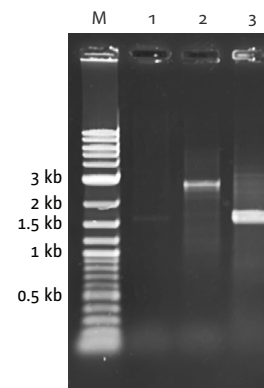

**C**

Distance  
from *oriC*  
[Mbp]

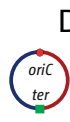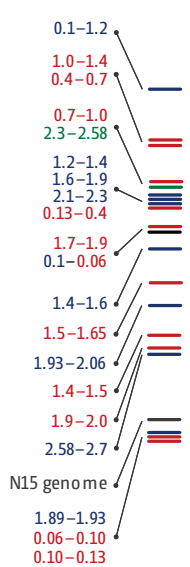

**D**

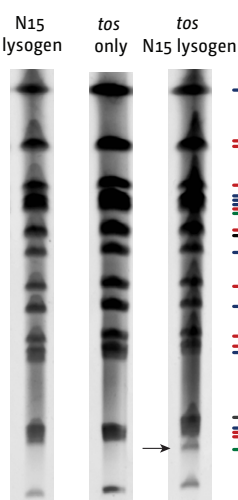

**E**

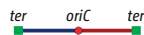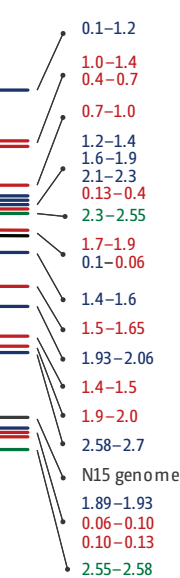

**F**

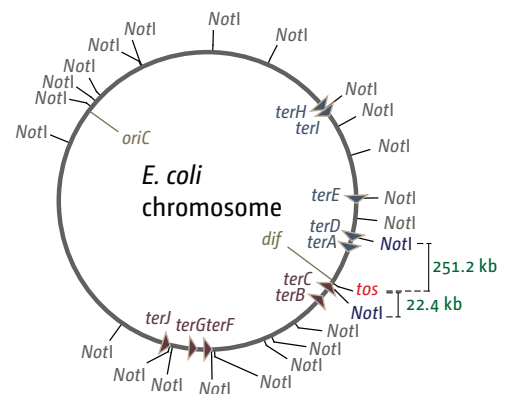

Supplement: Figure S1 — Verification of chromosome linearization in dnaA rnhA tos N15 lysogen cells. (A) Schematic representation of the area around dif with and without integrated tos-kan site. The linearization verification primers are shown in green (for primer sequences, see reference 12), and the PCR product sizes in wild-type cells and integrants are indicated. (B) PCR products generated with the linearization verification primers for tos-kan cells lysogenized with phage N15 (RCe403 [lane 1]), tos-kan cells (RCe401 [lane 2]), and an N15 lysogen of dnaA rnhA cells without the tos-kan linearization site (RCe383 [lane 3]). The shift of the PCR product size in a nonlinearized strain as shown in lane 2 indicates the presence of the tos-kan cassette. Linearization of the chromosome (lane 1) prevents formation of a specific PCR product since the chromosome is cleaved between the primer binding sites. The absence of a detectable PCR product confirms that the amount of circular chromosomes unprocessed by TelN in the population is very low, as reported previously (12). (C to F) Verification of chromosome linearization by pulsed-field gel electrophoresis (PFGE). If the tos site is cleaved by TelN, an additional band becomes visible on PFGE gels. The tos site is located in the 273.6-kb NotI fragment between positions 1337601 and 1611219 (C [highlighted in green]), and cleavage by TelN splits it into two fragments, one of which is 251.2 kb and the other of which is 22.4 kb (E and F [highlighted in green]). The 251.2-kb fragment moves into the quadruplet around 250 kb and thus is hidden in between other fragments (E). The smaller 22.4-kb fragment, however, becomes visible as an additional fragment at the bottom of the gel highlighted by a black arrow (D and E). A negative image is shown for clarity. Chromosomal DNA was prepared from RCe607 (rnhA N15 lysogen), RCe605 (rnhA tos-kan), and RCe608 (rnhA tos-kan N15 lysogen). Download [file mbo005152518sf1.pdf]

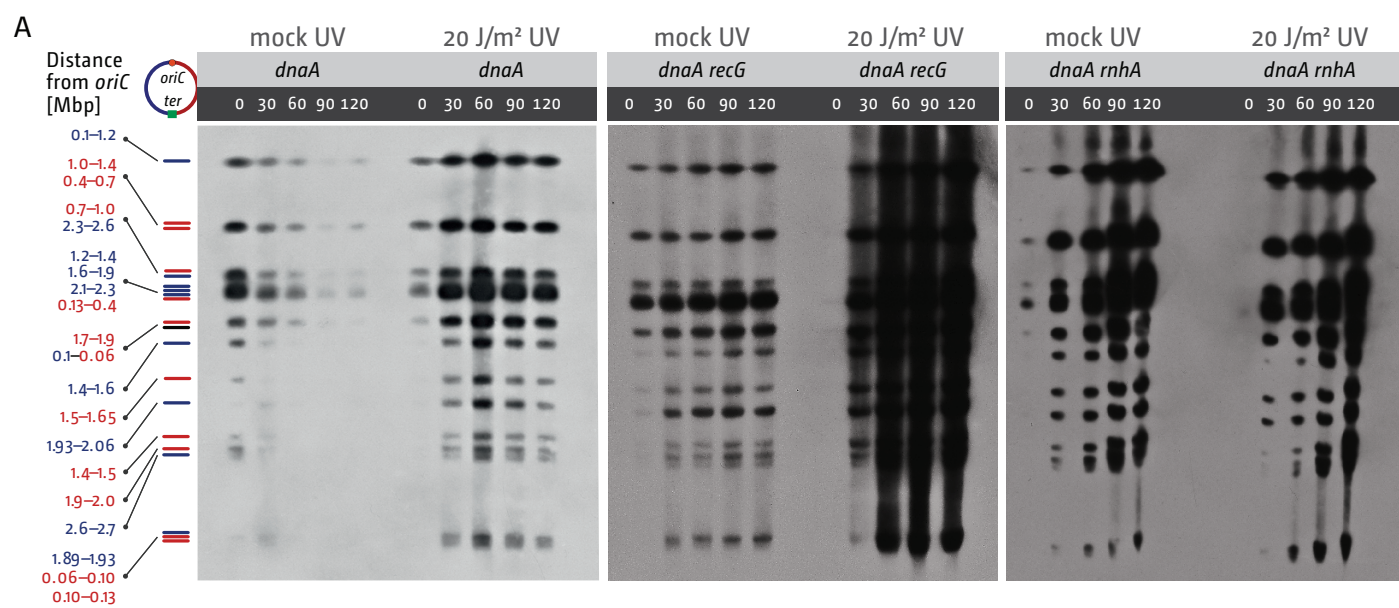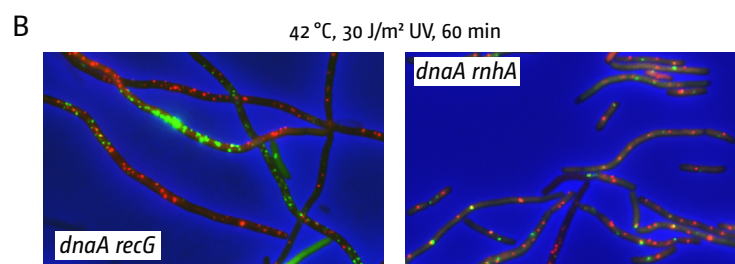

Supplement: Figure S2 — Damage-induced synthesis in cells lacking RNase HI. (A) Fluorograph showing a side-by-side comparison of BrdU incorporation into the chromosome of irradiated and mock-irradiated dnaA rnhA cells (AU1066). A schematic NotI restriction pattern of the E. coli chromosome is shown on the left, indicating the distance from oriC to each end of the shown fragments. Fragments clockwise and anticlockwise of oriC are shown in red and blue, respectively. Data for irradiated and mock-irradiated dnaA (AU1054) and dnaA recG (AU1091) cells were reproduced from reference 9 for comparison. The experiments were performed under comparable conditions on the same equipment. (B) Fluorescence microscopy showing replication of origin (red foci) and terminus (green foci) areas of the chromosome (combined phase-contrast and fluorescence images are shown) following the shift to the restrictive temperature in UV-irradiated cells. The strains used were AU1091 (dnaA recG) and AU1066 (dnaA rnhA). The incubation time after irradiation is indicated. Download [file mbo005152518sf2.pdf]

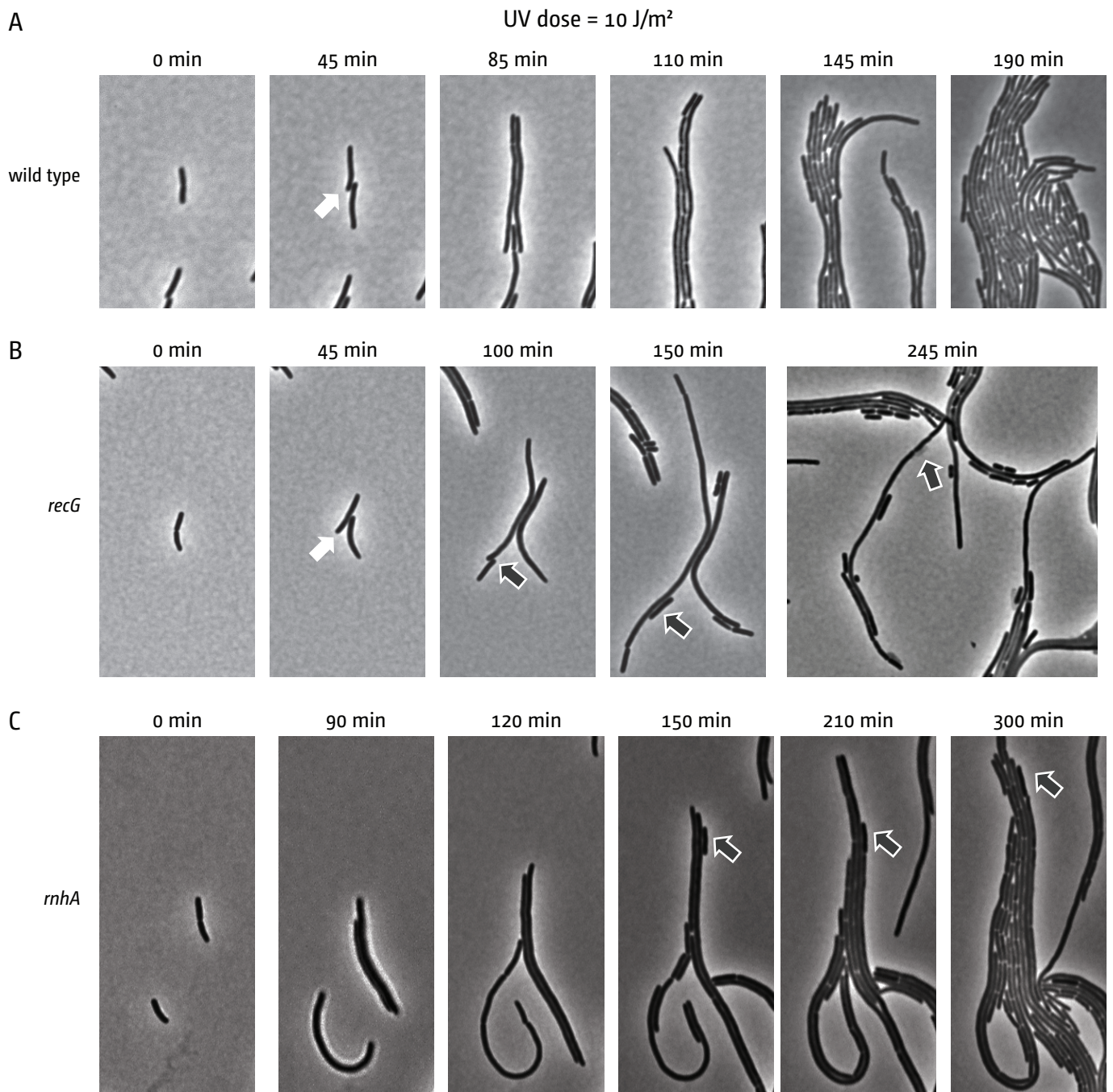

Supplement: Figure S3 — Effect of RNase HI on cell cycle progression of UV-irradiated cells. (A to C) Time-lapse photography following growth of single cells after UV irradiation. The strain used was N4704 (rnhA). Images of MG1655 (wild type) and N4560 (recG) were reproduced from reference 7 for comparison. White arrows indicate last divisions after irradiation before cells start to filament. Dark arrows illustrate dead cells budded off recG and rnhA filaments either showing no further divisions or bursting, leaving a “ghost.” While there is some extended filamentation in rnhA cells, the later time points clearly show that the filaments formed break up into small and normally growing cells. Experiments were performed under comparable conditions with the same equipment. Download [file mbo005152518sf3.pdf]

A

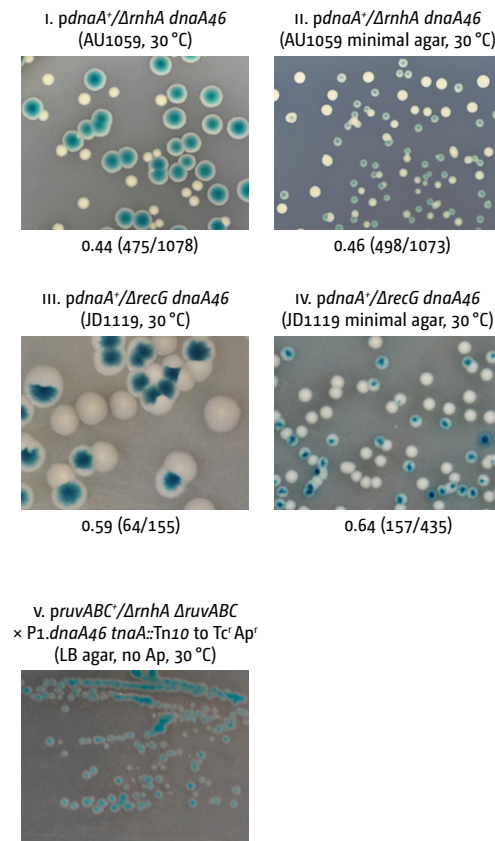

B

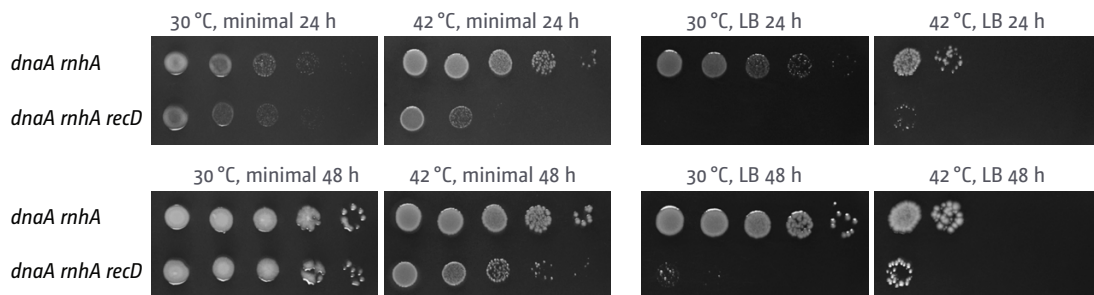

Supplement: Figure S4 — Effect of recD and ruvABC on cell survival and growth of cells lacking RNase HI. (A) Maintenance of cell viability in dnaA rnhA and dnaA rnhA ruv cells. The plasmids used were pAU101 (dnaA+) and pAM390 (ruvABC+) (for plasmids, see Text S1 in the supplemental material). Panels 1 to 4 show plate photographs of synthetic lethality assays, as described in Materials and Methods. The relevant genotype of the construct used is shown above each photograph, with the strain number in parentheses. The fraction of white colonies is shown below with the number of white colonies/total colonies analyzed in parentheses. For panel v, a transductant of a pruvABC+/ruvABC rnhA dnaA cross was streaked to single colonies on plates containing X-Gal/IPTG without ampicillin. (B) Spot dilution assays to evaluate origin-independent growth in dnaA rnhA cells in the absence of RecD. The strains used were AU1066 (dnaA rnhA) and JD1081 (dnaA rnhA recD). Download [file mbo005152518sf4.pdf]

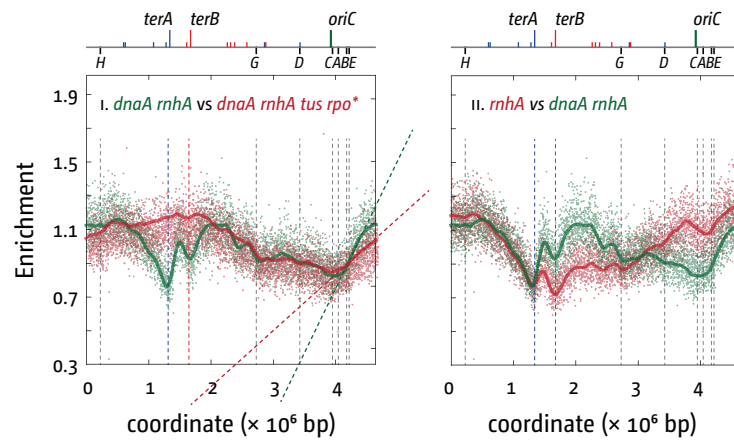

Supplement: Figure S5 — Overlay of replication profiles of rnhA derivatives. (A) Comparison of the replication profiles of dnaA rnhA and dnaA rnhA tus rpo* cells. Introduction of an rpo* point mutation changes the “step” observed at the position of the rrnCABE operon cluster, as indicated by dotted lines. The data sets are reproduced from Fig. 1. (B) Comparison of the replication profiles of rnhA and dnaA rnhA cells. The data sets are reproduced from Fig. 1. Download [file mbo005152518sf5.pdf]
