# Supplementary material for: The Consequences of Replicating in the Wrong Orientation: Bacterial Chromosome Duplication without an Active Replication Origin
Source: mBio. 2015 Nov 3;6(6):e01294-15. doi: 10.1128/mBio.01294-15 (PMC4631800; doi:10.1128/mBio.01294-15)
Supplement: Table S1 — List of all Escherichia coli K-12 constructs used in this study. [file mbo005152518st1.docx]

# Supplementary Table

## Table S1. Escherichia coli K-12 strains

| Strain | Relevant Genotype^a^ | Source^c^ |
| --- | --- | --- |
| **(a) General P1 donors** | | |
| RUC663 | *tnaA::*Tn*10 dnaA46* | Tove Atlung |
| **(b) MG1655 derivatives^b^** | | |
| MG1655 | F– *rph-1* | (13) |
| AM1578 | *ΔlacIZYA recB270::kan* pAM375 | A.A. Mahdi & R.G. Lloyd, unpublished |
| AM1660 | *ΔlacIZYA recA269::*Tn*10* pAM383 | (3) |
| AM1666 | *ΔrecA::apra* | (3) |
| AM1672 | *ΔrecD::dhfr* | (3) |
| AM1675 | *ΔrecB::dhfr* | A.A. Mahdi & R.G. Lloyd, unpublished |
| AM1975 | *ΔrnhA::apra* | A.A. Mahdi & R.G. Lloyd, unpublished |
| AM2304 | *ΔlacIZYA ΔproB::rnhA^+^-frt>kan>frt ΔrecG::apra* | (14) |
| APS345 | *att*Tn*7::lacO240-kan zdd/e::tetO240-gen* | (8) |
| AS1110 | *ΔlacIZYA dnaA46 tna::*Tn*10 recG265::cat* | AU1053 × P1.N4560 to Cm^r^ |
| AU1009 | *ΔlacIZYA* pJJ100 | TB28 × pJJ100 to Ap^r^ |
| AU1015 | *ΔlacIZYA recG::apra* | Plasmid-free derivative of JJ1119 |
| AU1018 | *ΔlacIZYA recA269::*Tn*10 rnhA::cat* pAM383 | AM1660 × P1.N4704 to Cm^r^ Ap^r^ |
| AU1020 | *ΔlacIZYA rnhA::cat* pJJ100 | AU1009 × P1.N4704 to Cm^r^ |
| AU1033 | *priA300 ΔlacIZYA rnhA::cat* pJJ100 | JJ1076 × P1.N4704 to Cm^r^ |
| AU1053 | *ΔlacIZYA dnaA46 tnaA::*Tn*10* | TB28 × P1.RUC663 to Tc^r^ |
| AU1054 | *dnaA46 tnaA::*Tn*10* | (15) |
| AU1057 | *ΔlacIZYA dnaA46 tnaA::*Tn*10* pAU101 | AU1053 × pAU101 to Ap^r^ |
| AU1059 | *ΔlacIZYA dnaA46 tnaA::*Tn*10 rnhA::cat* pAU101 | AU1057 × P1.N4704 to Cm^r^ |
| AU1066 | *ΔlacIZYA tnaA::*Tn*10 dnaA46 rnhA::cat* | (15) |
| AU1091 | *tnaA::*Tn*10 dnaA46 ΔrecG263::kan* | (15) |
| AU1191 | *ΔlacIZYA ΔruvABC::apra* | (16) |
| JD1041 | *ΔlacIZYA rnhA::cat* | plasmid-free derivative of AU1020 |
| JD1044 | *ΔlacIZYA rnhA::cat ΔrecD::dhfr* | JD1041 × P1.RCe304 to Tm^r^ |
| JD1051 | *ΔlacIZYA rnhA::cat ΔrecD::dhfr* pAM490 | JD1044 × pAM490 to Ap^r^ |
| JD1064 | *rpoB*35 ΔlacIZYA rnhA::cat* | N5925 × P1.JD1041 to Cm^r^ |
| JD1065 | *rpoB*35 ΔlacIZYA rnhA::cat* pAM490 | JD1064 ×pAM490 to Ap^r^ |
| JD1067 | *rpoB*35 ΔlacIZYA Δrep::cat rnhA::apra* pAM403 | JD1062 × P1.AM1975 to Apra^r^ Ap^r^ |
| JD1069 | *rpoB*35 ΔlacIZYA rnhA::cat recB::dhfr* pAM490 | JD1065 × P1.AM1675 to Tm^r^ Ap^r^ |
| JD1072 | *ΔlacIZYA rnhA::cat* pAM490 | JD1041 × pAM490 to Ap^r^ |
| JD1076 | *ΔlacIZYA rnhA::cat ΔrecD::dhfr dnaA46 tnaA::*Tn*10* pAM490 | JD1051 × P1.RUC663 to Tc^r^ |
| JD1077 | *ΔlacIZYA rnhA::cat ΔrecB::dhfr* pAM490 | JD1072 ×P1.AM1675 to Tm^r^ Ap^r^ |
| JD1081 | *ΔlacIZYA rnhA::cat ΔrecD::dhfr dnaA46 tnaA::*Tn*10* | plasmid-free derivative of JD1076 |
| JD1085 | *priA300 ΔlacIZYA rnhA::cat* | plasmid-free derivative of AU1033 |
| JD1089 | *rpoB*35 dnaA46 tnaA::*Tn*10* | N4849 × P1.RUC663 to Tc^r^ |
| JD1090 | *rpoB*35 dnaA46 tnaA::*Tn*10 recD::dhfr* | JJ1089 × P1.RCe304 to Tm^r^ |
| JD1104 | *ΔlacIZYA srgA1 argE::*Tn*10* | JJ1264 × P1.RCe300 to Tc^r^ |
| JD1107 | *ΔlacIZYA srgA1 rpoB*35* | JD1104 × P1.RCe395 to Tc^s^ Arg^+^ |
| JD1108 | *rpoB*35 dnaA46 tnaA::*Tn*10 recD::dhfr* pAM490 | JD1090 × pAM490 to Ap^r^ |
| JD1110 | *rpoB*35 ΔlacIZYA rnhA::cat ruvABC::apra* pAM490 | JD1065 × P1.AU1191 to Apra^r^ Ap^r^ |
| JD1113 | *rpoB*35 dnaA46 tnaA::*Tn*10 recD::dhfr rnhA::cat* pAM490 | JD1108 × P1.JD1041 to Cm^r^ Ap^r^ |
| JD1114 | *rpoB*35 ΔlacIZYA rnhA::cat ruvABC::apra  dnaA46 tnaA::*Tn*10* pAM490 | JD1110 × P1.RUC663 to Tc^r^ Ap^r^ |
| JD1119 | *ΔlacIZYA dnaA46 tnaA::*Tn*10 recG265::cat* pAU101 | AS1110 × pAU101 to Ap^r^ |
| JD1152 | *priA300 rpoB*35 dnaA46 tnaA::*Tn*10* | N5535 × P1.RUC663 to Tc^r^ |
| JD1153 | *ΔlacIZYA srgA1 rpoB*35 dnaA46 tnaA::*Tn*10* | JD1107 × P1.RUC663 to Tc^r^ |
| JD1156 | *priA300 rpoB*35 dnaA46 tnaA::*Tn*10 rnhA::cat* | JD1152 × P1.JD1041 to Cm^r^ |
| JD1158 | *ΔlacIZYA srgA1 rpoB*35 dnaA46 tnaA::*Tn*10 rnhA::cat* | JD1153 × P1.JD1041 to Cm^r^ |
| JD1160 | *rpoB*35 tnaA::*Tn*10 dnaA46 rnhA::cat tus1::dhfr  tos-kan* | RCe309 × P1.RCe427 to Km^r^ |
| JD1162 | *priA300 rpoB*35 dnaA46 tnaA::*Tn*10 rnhA::cat tus1::dhfr* | JD1156 × P1.N6798 to Tm^r^ |
| JD1163 | *ΔlacIZYA srgA1 rpoB*35 dnaA46 tnaA::*Tn*10 rnhA::cat tus1::dhfr* | JD1158 × P1.N6798 to Tm^r^ |
| JD1165 | *ΔlacIZYA srgA1 rpoB*35 dnaA46 tnaA::*Tn*10 recG::apra* | JD1153 × P1.AU1015 to Apra^r^ |
| JD1167 | *ΔlacIZYA srgA1 rpoB*35 dnaA46 tnaA::*Tn*10 recG::apra tus1::dhfr* | JD1165 × P1.N6798 to Tm^r^ |
| JD1168 | *rpoB*35 tnaA::*Tn*10 dnaA46 rnhA::cat tus1::dhfr* N15 lysogen | RCe309 × N15 to N15^r^ |
| JD1169 | *rpoB*35 tnaA::*Tn*10 dnaA46 rnhA::cat tus1::dhfr  tos-kan* N15 lysogen | JD1160 × N15 to N15^r^ |
| JJ1119 | *ΔlacIZYA recG::apra* pJJ100 | (17) |
| JJ1060 | *priA300 ΔlacIZYA* | plasmid-free derivative of N5933 |
| JJ1076 | *priA300 ΔlacIZYA* pJJ100 | JJ1060 × pJJ100 to Ap^r^ |
| JJ1264 | *ΔlacIZYA srgA1* | J. Zhang & R.G. Lloyd, unpublished |
| JJ1359 | *ΔlacIZYA dam1::kan recG::apra tus1::dhfr* | J. Zhang & R.G. Lloyd, unpublished |
| MGK297 | *att*Tn*7::tetO240-gen zdd/e::lacO240-cat tos-kan* | (12) |
| N4560 | *recG265::cat* | (3) |
| N4704 | *rnhA::cat* | (16) |
| N4837 | *argE::*Tn*10* | (3) |
| N4849 | *rpoB*35* | (3) |
| N5494 | *Δtus::kan* | (15) |
| N5535 | *priA300 rpoB*35* | (3) |
| N5925 | *rpoB*35 ΔlacIZYA* | R.G. Lloyd, unpublished |
| N5933 | *priA300 ΔlacIZYA* pAM374 | (3) |
| N6796 | *tus1::dhfr* | (15) |
| N6798 | *recG265::cat tus1::dhfr* | N4560 × P1.JJ1359 to Tm^r^ |
| RCe072 | *att*Tn*7::lacO240-kan zdd/e::tetO240-gen recG265::cat* | (7) |
| RCe197 | *att*Tn*7::lacO240-kan zdd/e::tetO240-gen  tnaA::*Tn*10 dnaA46* | APS345 × P1.RUC663 to Tc^r^ |
| RCe198 | *att*Tn*7::lacO240-kan zdd/e::tetO240-gen recG265::cat tnaA::*Tn*10 dnaA46* | RCe072 × P1.RUC663 to Tc^r^ |
| RCe202 | *att*Tn*7::lacO240-kan zdd/e::tetO240-gen  tnaA::*Tn*10 dnaA46 rnhA::cat* | RCe197 × P1.N4704 to Cm^r^ |
| RCe218 | *ΔlacIZYA tnaA::*Tn*10 dnaA46 rnhA::cat Δtus::kan* | AU1066 × P1.N5494 to Km^r^ |
| RCe262 | *rpoB*35 tnaA::*Tn*10 dnaA46* | N4849 × P1.RUC663 to Tc^r^ |
| RCe268 | *rpoB*35 ΔrecG::apra Δtus::cat dnaA46 tnaA::*Tn*10* | (15) |
| RCe300 | *att*Tn*7::lacO240-kan zdd/e::tetO240-gen argE::*Tn*10* | APS345 × P1.N4837 to Tc^r^ Arg^–^ |
| RCe303 | *rpoB*35 tnaA::*Tn*10 dnaA46 rnhA::cat* | RCe262 × P1.N4704 to Cm^r^ |
| RCe304 | *att*Tn*7::lacO240-kan zdd/e::tetO240-gen recD::dhfr* | APS345 × P1.AM1672 to Tm^r^ |
| RCe309 | *rpoB*35 tnaA::*Tn*10 dnaA46 rnhA::cat tus1::dhfr* | RCe303 × P1.N6796 to Tm^r^ |
| RCe313 | *priA300 rpoB*35 recG::apra tus::cat  tnaA::*Tn*10 dnaA46* | (15) |
| RCe326 | *rpoB*35 ΔrecG::apra Δtus::cat dnaA46 tnaA::*Tn*10* pDIM104 | RCe268 × pDIM104 to Ap^r^ |
| RCe383 | *ΔlacIZYA tnaA::*Tn*10 dnaA46 rnhA::cat* N15 lysogen | AU1066 × N15 to N15^r^ |
| RCe384 | *rpoB*35 recG::apra tus::cat tnaA::*Tn*10 dnaA46* N15 lysogen | (15) |
| RCe385 | *rpoB*35 recG::apra tus::cat tnaA::*Tn*10 dnaA46  tos-kan* | (15) |
| RCe387 | *rpoB*35 recG::apra tus::cat tnaA::*Tn*10 dnaA46  tos-kan* N15 lysogen | (15) |
| RCe395 | *rpoB*35 tnaA::*Tn*10 dnaA46 rnhA::cat tus1::dhfr ΔoriC::kan* | (15) |
| RCe401 | *ΔlacIZYA tnaA::*Tn*10 dnaA46 rnhA::cat tos-kan* | AU1066 × P1.MGK297 to Km^r^ |
| RCe403 | *ΔlacIZYA tnaA::*Tn*10 dnaA46 rnhA::cat tos-kan* N15 lysogen | RCe401 × N15 to N15^r^ |
| RCe427 | *tos-kan* | MG1655 × P1.MGK297 to Km^r^ |
| RCe529 | *rpoB*35 tnaA::*Tn*10 dnaA46 rnhA::cat tus1::dhfr ΔrecA::apra* | RCe309 × P1.AM1666 to Apra^r^ |
| RCe552 | *ΔlacIZYA tna::*Tn*10 dnaA46 rnhA::cat* pECR22 | AU1066 × pECR22 to Ap^r^ |
| RCe557 | *ΔlacIZYA tna::*Tn*10 dnaA46 rnhA::cat* pLau17 | AU1066 × pLau17 to Ap^r^ |
| RCe605 | *tos-kan rnhA::apra* | RCe427 × P1.AM1975 to Apra^r^ |
| RCe607 | *rnhA::apra* N15 lysogen | AM1975 × N15 to N15^r^ |
| RCe608 | *tos-kan rnhA::apra* N15 lysogen | RCe605 × N15 to N15^r^ |
| RCe615 | *priA300 ΔlacIZYA rnhA::cat tnaA::Tn10 dnaA46* | JD1085 × P1.RUC663 to Tc^r^ |
| SLM1008 | *rpoB*35 ΔrecG::apra Δtus::cat tnaA::*Tn*10 dnaA46* pECR22 | RCe268 × pECR22 to Ap^r^ |
| SLM1010 | *rpoB*35 ΔrecG::apra Δtus::cat tnaA::*Tn*10 dnaA46* pLau17 | RCe268 × pLau17 to Ap^r^ |
| SLM1104 | *ΔlacIZYA tnaA::*Tn*10 dnaA46 rnhA::cat* pDIM104 | AU1066 × pDIM104 to Ap^r^ |
| TB28 | *ΔlacIZYA<>frt* | (10) |

a – The abbreviations *kan*, *cat*, *dhfr, gen* and *apra* refer to insertions conferring resistance to kanamycin (Km^r^), chloramphenicol (Cm^r^), trimethoprim (Tm^r^), gentamicin (Gm^r^) and apramycin (Apra^r^), respectively. Tn*10* confers resistance to tetracycline (Tc^r^). Plasmids carry an ampicillin (Amp^r^) resistance marker. Strains carrying *dnaA46* are temperature sensitive for growth. *ΔoriC* refers to a replacement of the entire origin region (754 bp) including DnaA boxes and 13mers as well as the entire *mioC* gene by a kanamycin resistance cassette (15). *tos-kan* refers to the telomerase occupancy site from the bacteriophage N15 genome followed by a kanamycin resistance cassette (12).

b – Only the relevant additional genotype of the derivatives is shown. For all relevant plasmids see Supplementary Material.

c – The term “× N15 to N15^r^” refers to isolation of *E. coli* cells lysogenized with bacteriophage N15. These cells can be identified by their resistance to re-infection with N15.
